# GEN-Click: Genetically Encodable Click Reactions for Spatially Restricted Metabolite Labeling

**DOI:** 10.1021/acscentsci.3c00511

**Published:** 2023-07-25

**Authors:** Pratyush Kumar Mishra, Nirmali Sharma, Hyunwoo Kim, Changwook Lee, Hyun-Woo Rhee

**Affiliations:** † Department of Chemistry, Seoul National University, Seoul 08826, Republic of Korea; ‡ Department of Biological Sciences, Ulsan National Institute of Science and Technology, Ulsan 44919, Republic of Korea; § School of Biological Sciences, Seoul National University, Seoul 08826, Republic of Korea

## Abstract

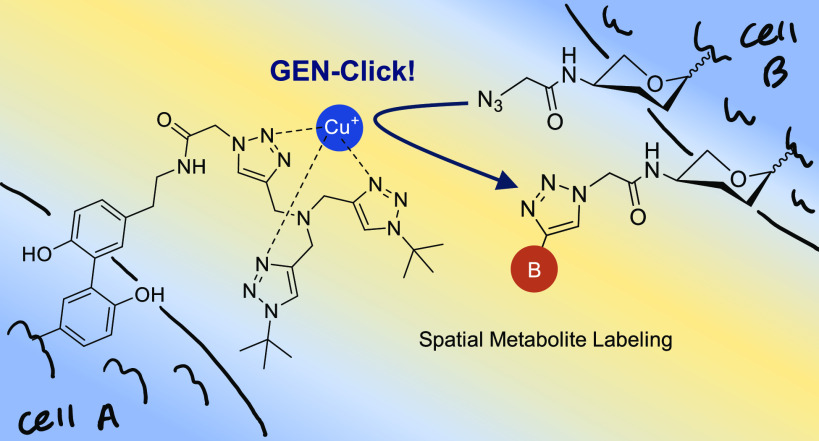

Chemical reactions for the *in situ* modification of biomolecules within living cells are under development. Among these reactions, bio-orthogonal reactions such as click chemistry using copper(I) and Staudinger ligation are widely used for specific biomolecule tracking in live systems. However, currently available live cell copper(I)-catalyzed azide/alkyne cycloaddition reactions are not designed in a spatially resolved manner. Therefore, we developed the “GEN-Click” system, which can target the copper(I)-catalyzed azide/alkyne cycloaddition reaction catalysts proximal to the protein of interest and can be genetically expressed in a live cell. The genetically controlled, spatially restricted, metal-catalyzed biorthogonal reaction can be used for proximity biotin labeling of various azido-bearing biomolecules (e.g., protein, phospholipid, oligosaccharides) in living cell systems. Using GEN-Click, we successfully detected local metabolite-transferring events at cell–cell contact sites.

## Abstract 2

GEN-Click is a genetically encoded copper-click reaction. It enables spatial biotin labeling of the azide or alkyne-tagged biomolecules in live cells.

## Introduction

The copper(I)-catalyzed azide/alkyne cycloaddition (CuAAC) reaction can be used to study the chemical biology of metabolites in live systems.^1,2^ Surrogate biomolecules bearing small functional groups (e.g., azidohomoalanine (AHA),^3^ azido-sugars,^4^ and alkyne-choline^5^) can be incorporated into more complex biological systems via a normal metabolic pathway and then visualized or selectively enriched via the CuAAC reaction for further analysis. For these chemical biology studies, various triazole-based ligands (e.g., THPTA, BTTA^6^) that stabilize Cu(I) ions in the aqueous environments of biological systems have been developed, leading to efficient reaction kinetics for various biological molecules.^6^

**Figure 1:**
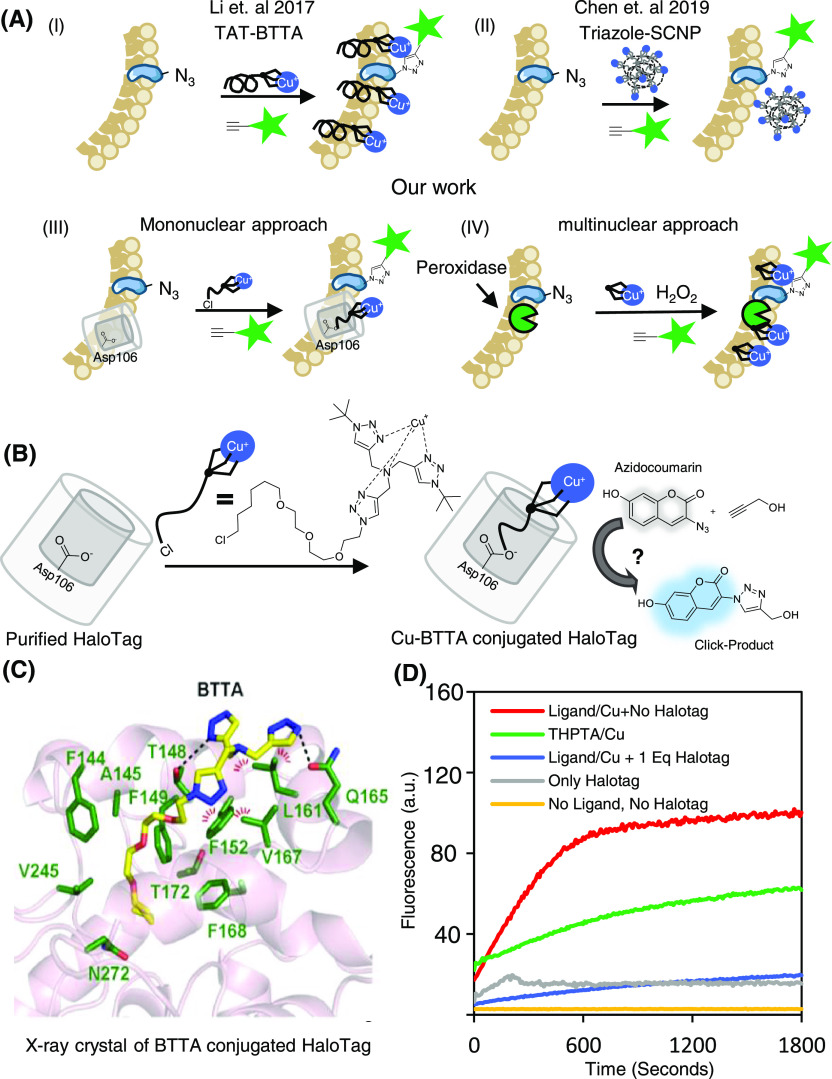
HaloTag-based GEN-Click approach. (A) Schematic representation of various catalytic CuAAC reactions in live cells: (i) TAT-BTTA conjugate peptide approach, (ii) Cu(I)-binding single-chain nanoparticle-based approach, (iii) HaloTag-based mononuclear copper-click approach (approach #1), and (iv) multinuclear peroxidase-based copper-click approach (approach #2). (B) Scheme of fluorogenic CuAAC reaction of BTTA-PEG3-HTL with HaloTag and azidocoumarin. (C) X-ray crystal structure of the holo-protein complex of BTTA-HTL:HaloTag (PDB ID 8J1O). (D) Fluorogenic CuAAC reaction between free BTTA-PEG3-HTL (10 μM) and BTTA-PEG3-HTL:HaloTag complex (10 μM). 10 μM CuSO_4_, 50 μM azidocoumarin, 100 μM propargyl alcohol, and 2.5 mM sodium ascorbate were added.

Surrogate biomolecules can be globally incorporated into a newly synthesized proteome/metabolome.^7−9^ Thus, stabilizing Cu(I) ions in the aqueous environments of biological systems permits efficient reaction kinetics of various biological actions.^6,10,11^ For example, the artificial metalloenzyme “clickase”, which is based on Cu(I)-binding single-chain nanoparticles^12^ and the cell-permeable Cu-binding TAT peptide-conjugated BTTA,^13^ can be used to perform CuAAC reactions in live cells (Figure 1A); however, they do not exhibit spatially controlled reactions at designated sites. Therefore, we aimed to develop a genetically encodable click reaction (GEN-Click) system that can selectively target the CuAAC reaction catalyst proximal to the protein of interest and that can be genetically expressed in live cells. To selectively tag the Cu(I)-binding catalyst on the protein of interest, we employed two different methods for protein modification using genetic tag proteins. One method used the HaloTag,^14−16^ which can be selectively conjugated with chloroalkane-conjugated ligands. The other method used peroxidase (APEX2), which can modify proximal tyrosine (Tyr) residues with phenol-conjugated probes within 20 nm in live cells.^17^ Using these systems, we targeted the Cu(I)-BTTA catalyst to proteins of interest and tested their CuAAC reactions with proximal azido-conjugated biomolecules in live cells (Figure 1A).

## Results and Discussion

To test our GEN-Click approach using HaloTag (Figure 1B), we synthesized a chloroalkane-based HaloTag ligand (HTL) conjugated to a BTTA moiety connected by poly(ethylene glycol) (PEG) linkers of various lengths (BTTA-PEG_*n*_-HTL, Scheme S1). Our BTTA-PEG-HTL ligands exhibited favorable HaloTag binding properties, as confirmed in a competitive binding assay (Figure S1). Additionally, we characterized the X-ray crystal structure of the BTTA-HTL (PDB ID 8J1O, Figure 1C):HaloTag protein complex and observed that the BTTA moiety of BTTA-PEG3-HTL was well-exposed at the protein surface. However, the BTTA-HTL ligand did not show high CuAAC catalytic activity for azidocoumarin and propargyl alcohol as a substrate when bound to HaloTag, despite showing good catalytic activity in the free form (Figure 1D and Figure S2). Our crystal data suggest that the specific surface residues of HaloTag strongly inhibit copper complexation of BTTA via π–π or hydrogen-bonding events at the surface (Figure 1C).

**Figure 2:**
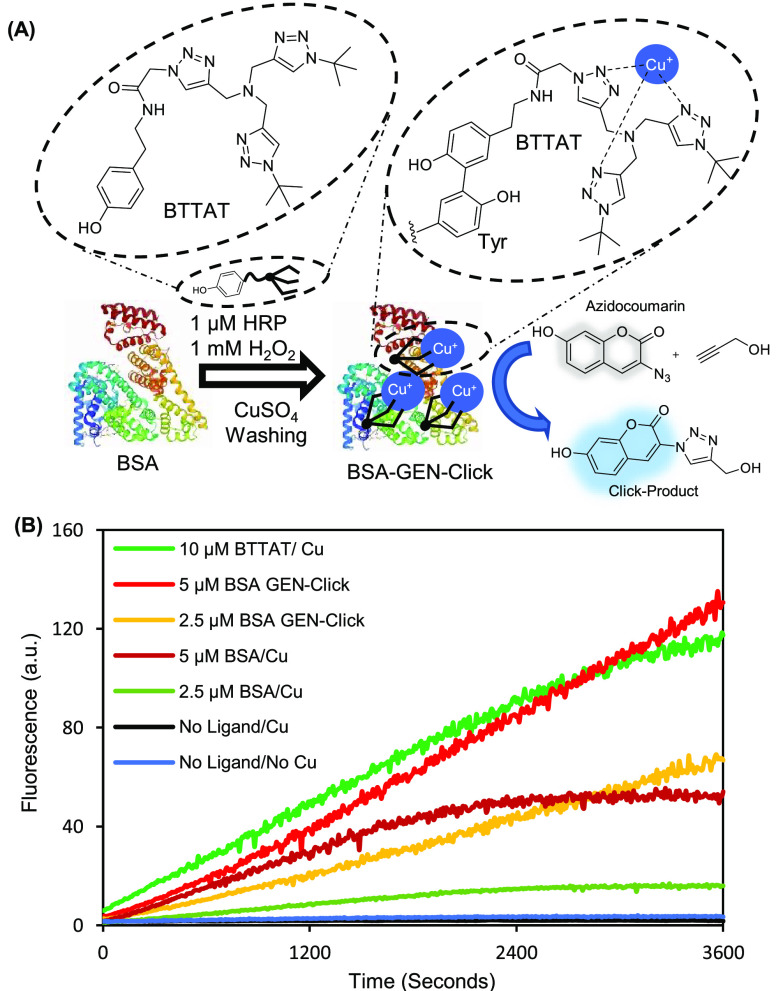
Peroxidase-based GEN-Click reaction. (A) Schematic representation of peroxidase-mediated conversion of protein to the GEN-Click catalyst via BTTAT modification. For the model *in vitro* reaction, horseradish peroxidase (HRP) and bovine serum albumin (BSA) were used as the peroxidase and substrate protein, respectively (see Synthesis Protocol in the Supporting Information). (B) Fluorogenic CuAAC reaction monitoring results of BSA-GEN-Click (Cu/BTTAT-modified BSA) and controls (e.g., Cu-BSA, Cu-BTTAT, and no ligand). Briefly, 50 μM azidocoumarin, 100 μM propargyl alcohol, and 2.5 mM sodium ascorbate were added to the catalyst, and the fluorescence was measured at 477 nm (excitation at 404 nm).

Based on this result, we utilized a peroxidase-mediated proximity labeling reaction to covalently conjugate phenol-conjugated probes to proximal Tyr residues within a 20 nm labeling radius.^18^ We hypothesized that BTTA-conjugated tyramide (BTTAT) would react with genetically encoded peroxidase enzymes (i.e., APEX^19^ or horseradish peroxidase (HRP)^20^) to generate BTTAT-conjugated Tyr residues, which acted as coordinating ligands for Cu(I) catalysts to facilitate the CuAAC reaction at the designated site (Figure 2A).

To test our hypothesis, we synthesized BTTAT (Scheme S1) and conducted fluorescence-based monitoring of its CuAAC catalytic activity. We observed effective catalysis by BTTAT (Figure S2D), as compared to that of THTPA and BTTAA.^6^ Additionally, we evaluated whether BTTAT catalyzed the CuAAC reaction when conjugated with proteins. For the test tube reaction, we utilized HRP as a peroxidase catalyst to modify BTTAT conjugation to the Tyr residues of bovine serum albumin (BSA), which can be modified by phenoxyl radicals generated by HRP.^21^ The catalytic activity of BTTAT-modified BSA was 2.5-fold higher than that of nonmodified BSA, although the basal activity of BSA with copper might be due to the metal binding sites present within BSA (Figure 2).^22^ We examined the catalytic activity of BTTAT-modified BSA *in vitro* using the azidohomoalanine (AHA)-labeled cell lysate in reaction with desthiobiotin-alkyne. We found that BTTAT-modified BSA generated numerous desthiobiotin-conjugated proteins via streptavidin-HRP Western blotting (Figure S3). These results indicate that BTTAT functions in the CuAAC reaction even when conjugated to the Tyr residues of BSA.

After demonstrating the CuAAC reaction of BTTAT *in vitro*, we designed an in-cell GEN-Click reaction of BTTAT using genetically expressed peroxidase in living cells. For this experiment, we transfected the APEX2-TM construct that can be genetically expressed at the cell surface and incubated HEK293T cells with mannose-azide (ManAz), which can be converted to azido-sialic acid and incorporated on the glycan through an existing metabolic pathway.^23^

**Figure 3:**
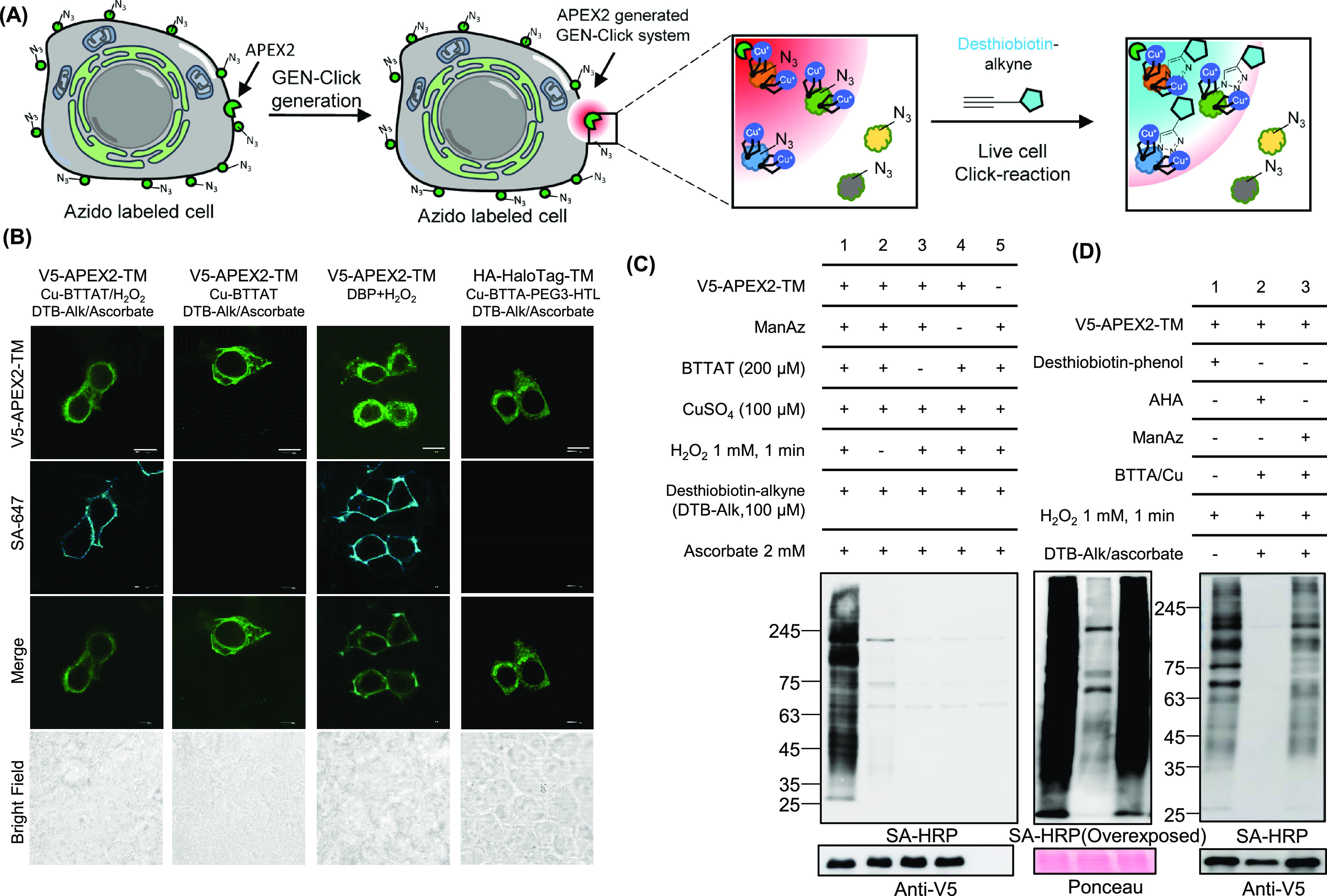
GEN-Click reactions for APEX2-TM expressed on the cell surface for glycan labeling. (A) Scheme of the APEX-driven GEN-Click reaction with azido-decorated biomolecules at the cell surface. (B) Confocal imaging results of the APEX-driven Cu-BTTAT labeling activity. Imaging results of APEX2:desthiobiotin-phenol labeling and HaloTag:desthiobiotin-alkyne (DTB-Alk) labeling, with BTTA-HTL results as controls for comparison. Expression levels of APEX2-TM were confirmed using anti-V5, and SA-647 was used to visualize desthiobiotin labeling. (C) Western blot results of APEX-driven Cu-BTTAT labeling activity. Omission of BTTAT, ManAz, or APEX-TM expression was used as control. (D) Comparison of Cu-BTTAT labeling signals with AHA- or ManAz-incorporated cells. All desthiobiotin-alkyne labeling reactions in (C) and (D) were conducted in live cells. Expression levels of APEX2-TM were confirmed using anti-V5, and streptavidin-horseradish peroxidase (SA-HRP) was used to visualize desthiobiotin labeling.

We next preincubated BTTAT (200 μM) and CuSO_4_ (100 μM) and applied the mixture to cells for 1 min in the presence of 1 mM H_2_O_2_ to generate BTTAT-modified Tyr residues at the transfected cell surface (Figure 3A). The catalytic reaction on Cu-BTTAT-modified cells was initiated with 100 μM desthiobiotin-alkyne and 2 mM sodium ascorbate incubation for 10 min; desthiobiotin-conjugated glycan, which is a product of this CuAAC reaction, can be visualized using streptavidin-AF647 fluorophore (SA-647) staining.

We detected selective desthiobiotin-alkyne labeling (SA-647 stain) on the surface of cells expressing APEX2-TM (Figure 3B and Figure S4). The desthiobiotin-alkyne conjugation intensity of GEN-Click is comparable to the desthiobiotin-phenol labeling intensity of APEX2-TM, although these two desthiobiotin labelings were generated on different biomolecules (e.g., glycan vs proteins). No desthiobiotin-alkyne labeling was observed when H_2_O_2_, BTTAT, or ManAz/AHA was omitted. Further, AHA-incorporated cells were modified through a CuAAC reaction of BTTAT-modified cells, even though the desthiobiotin-alkyne labeling intensity was lower than that of ManAz-treated cells (Figure 3D and Figure S4) possibly because of different solvent exposure levels at AHA- or ManAz-incorporated sites. As expected, HaloTag-TM expressing cells showed no desthiobiotin-alkyne labeling with BTTA-HTL (Figure 3B).

Notably, no APEX2-TM-expressing cells (nontransfected cells) in the same culture plate as APEX2-TM-transfected cells exhibited a desthiobiotin-alkyne labeling signal, although they were exposed to the same reagents (e.g., Cu-BTTAT and desthiobiotin-alkyne) under the same incubation conditions (Figure 3B). This result indicates that the CuAAC reaction with BTTAT is selectively driven by APEX2 and that the reaction is genetically controllable via APEX expression. The lack of desthiobiotin-alkyne labeling in the absence of H_2_O_2_ during Cu-BTTAT incubation shows that Cu-BTTAT modification can be precisely controlled by using the APEX2 activity.

Our GEN-Click reactions can be utilized in various applications with metabolically generated azide- or alkyne-functionalized biomolecules. For instance, we tested whether the GEN-Click reaction could be used to identify ligand–receptor interactions at the cell surface. For this experiment, we prepared a “ligand” protein, a secreted APEX2-conjugated frankenbody^24^ with binding affinity toward the HA epitope tag. We also prepared “recipient” cells by treating and transfecting the cells with ManAz and HA-mCherry-TM (a receptor protein for frankenbody), respectively.

Next, we administered the recipient cells with the ligand protein and incubated them with BTTAT/H_2_O_2_ molecules to generate GEN-Click moieties that were localized to APEX2. Subsequently, desthiobiotin-alkyne and sodium ascorbate were sequentially applied for selective biotin conjugation on the azido-glycan at the recipient cells. As expected, selective generation of the biotinylation signal occurred in the HA-mCherry-TM-expressing cells (detected using SA-647, Figure S5). We also further validated that GEN-Click showed more specific biotin labeling of the receptor protein, compared to the biotin-phenoxyl radical labeling of APEX2 (Figure S5D). This result confirmed that our GEN-Click reaction system can be used to identify ligand receptor pairs with therapeutic potential.

We also investigated whether the GEN-Click system can catalyze the proximal click reaction on alkyne-phospholipids. Alkyne-choline,^5^ which can be metabolically converted to phosphatidylcholine bearing the alkyne moiety on the outer leaflet of the cell membrane, was visualized on the cell surface phosphatidylcholine from the turn-on click signal using azidocoumarin using our GEN-Click reactions on a live cell surface (Figure S6). As current commercially available proximity labeling techniques (APEX, BioID/TurboID) cannot generate a biotin signal on glycan or lipid metabolites, our GEN-Click method expands the substrate spectrum from proteins to other metabolites.

**Figure 4:**
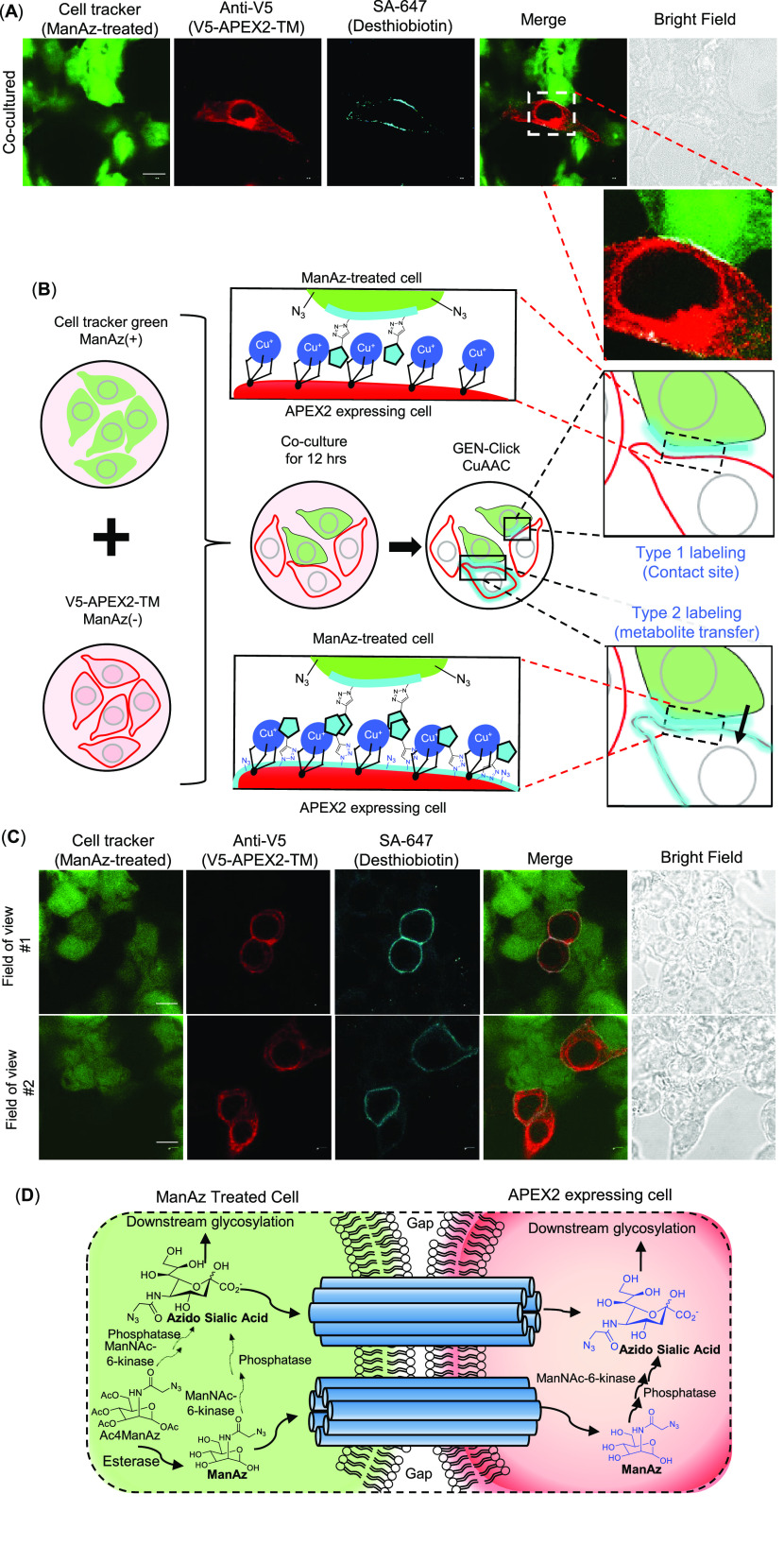
Cell–cell communication visualization using GEN-Click assisted biotinylation. (A) Confocal images of cell–cell contact site labeling by GEN-Click-assisted biotinylation of ManAz-labeled cell. (B) Schematic representation of GEN-Click-assisted cell–cell contact site labeling (type 1 labeling) and intercellular metabolite transfer labeling (type 2 labeling) in cocultured sample. The GEN-Click reaction can be used to label processed azide groups on the cell surface of the contact cell and on the surface of recipient cells in the event of metabolite transfer via cell contact. (C) Confocal images of cell–cell contact site and metabolite transfer labeling (type 2 labeling) in a cocultured sample of ManAz-treated cells (marked with Cell Tracker, green fluorescence) and V5-APEX2-TM expressing cells (marked with anti-V5/Mouse-568 antibody). Desthiobiotin-labeled molecules were visualized with Streptavidin-AF647 (SA-647). Scale bar: 10 μm. (D) Schematic representation of ManAz conversion and transfer to adjacent cells via the gap junction. Transferred metabolites were marked with blue in the schemes of (B) and (D).

Selective proximity labeling on the azide-bearing molecules enables the design of unique applications to identify metabolite transfer events at the cell–cell contact sites. In this experiment, we prepared “GEN-Click” cells (i.e., APEX2-TM transfected for BTTAT modification) and “azido-metabolite” cells (i.e., ManAz-treated). After growing the cells in separate dishes, we cocultured APEX2-TM-transfected cells with ManAz-treated cells (marked with Cell Tracker, Figure 4A) in the same dish. These cells formed cell–cell contact sites during the overnight coculturing. We next treated BTTAT/H_2_O_2_ for *in situ* generation of GEN-Click in APEX2-TM-transfected cells and added desthiobiotin-alkyne to label any azido-glycan contents proximal to GEN-Click. Using this approach, we detected ManAz-treated cells in direct contact that were biotin-conjugated by GEN-Click cells (Figure 4A), particularly at the cell–cell contact site (type 1 labeling in Figure 4B).

Interestingly, several APEX2-TM-expressing cells (no ManAz treatment) with a direct contact site with ManAz-treated cells showed a biotinylated signal at cell–cell contact sites and an evenly distributed biotinylated signal at the cell surface of the APEX2-TM cells (Figure 4B,C). For further confirmation, we examined APEX2-TM cells that were not in the proximity of ManAz-treated cells (as indicated by Cell Tracker), which revealed that the labeling pattern was absent (Figure S7A), supporting the transfer of ManAz or its metabolic-converted forms to APEX2-TM cells (Figure 4D). We also validated that a similar metabolite transfer labeling pattern was also observed in the cocultured sample of alkyne-choline treated cells and APEX2-TM expressing cells (Figure S7B,C).

Additionally, we confirmed that metabolite transfer requires direct cell–cell contact and could not occur through the medium (Figure S8A). We also observed that metabolite transfer labeling in the cocultured system was largely affected when the coculturing incubation time was changed from 12 to 4 h (Figure S8B). Treatment with 100 μM carbenoxolone, a well-known gap junction inhibitor,^25^ also affected the evenly distributed GEN-Click labeling pattern (type 2 labeling) on the APEX2-TM cells (Figure S8C). All of these results support the idea that GEN-Click can capture the metabolite transfer event through the gap junctions.

In this study, we developed a GEN-Click method to catalyze biorthogonal click reactions in a genetically encoded spatially localized manner in living cells. From a chemical perspective, our work significantly broadens the substrate scope of proximity labeling. In comparison to the currently limited range of substrates for proximity labeling (such as proteins, RNA, and DNA),^26^ the utilization of GEN-Click enables an expanded substrate scope. It allows for the labeling of azido-incorporated proteins (e.g., AHA) as well as azido-incorporated metabolites (such as glycans and phospholipids), utilizing various azido- or alkyne-bearing surrogate biomolecules that can be taken up via salvage pathways in live cells.^27^

From a biological standpoint, the expandable substrate scope of GEN-Click is also beneficial for monitoring various metabolite transfer events via the gap junction. Currently, only limited metabolites (e.g., ATP, NAD^+^, glutamate, glutathione, PGE2)^28^ have been characterized to be transferred at the cell–cell interface via gap junctions and our study can suggest that mannose and choline (or their derivatives) can be added to this list. We anticipate that our system can be utilized to monitor metabolite transfer events by using diverse surrogate biomolecules. Additionally, it can be employed for inhibitor screening of these events, which is known to hinder tumor survival pathways under mutagenesis conditions.^29^

As a proof-of-concept system, our GEN-Click with BTTAT can be used to perform the CuAAC reaction at a specific site on the cell surface (APEX2-TM-expressing cells); however, the low Cu(I)-binding affinity and poor membrane permeability of BTTAT may prevent its use in the cytosolic space. This limitation may be overcome by using a strong Cu(I)-binding motif.^30^ It is also noteworthy that our HaloTag-BTTA system could potentially be improved by engineering the HaloTag protein or ligand. This aspect presents an interesting topic in the artificial metalloenzyme field.^31^

## Materials and Methods

### Plasmids and Cloning

Genes were cloned into the specified vectors using standard enzymatic restriction digest and ligation with T4 DNA ligase. To generate constructs where short tags (e.g., V5 epitope or AviTag) or signal sequences were appended to the protein, we included the desired tag in the gene-specific primers used for PCR amplification. PCR products were digested with restriction enzymes and ligated into cut vectors (e.g., pcDNA3, pCDNA5, pDisplay, pET21a, and pH6HTN). In all cases, the cytomegalovirus promoter was used for expression in mammalian cells. See Table S2 for detailed information on the constructs.

### Protein Purification

Gene encoding protein was amplified using PCR and cloned into a modified pH6HTN vector with histidine tags (6X His). The genes were transformed into BL21 (DE3) *Escherichia coli* cells at 42 °C for 30 s, and cells were cultured on an ampicillin-treated agar plate. A colony was picked into 5 mL of ampicillin containing LB-Broth overnight, and 1 mL of LB was transferred to 1 L of new LB broth. After reaching 0.5 optical density, cells were treated with 0.25 mM isopropylthio-β-galactoside and cultured at 18 °C for 24 h. Cells were harvested 24 h postinduction, lysed by B-per buffer (Invitrogen), and centrifuged, and the protein was purified via Ni^2+^-NTA chromatography. The eluted protein was finally concentrated, and the free imidazole ring was removed by using an Amicon Ultra-15 centrifugal filter (*M*_w_, 10 kDa cutoff, Millipore) and flash-frozen in liquid nitrogen for storage. For crystallization, HaloTag proteins were purified as previously described.^21^ For the formation of HaloTag complexed with VL1 and UL2 ligands, purified proteins were mixed with 3-fold molar excesses of VL1 and UL2, respectively. After incubating for 3 h on ice, proteins were injected onto a size-exclusion column (GE Healthcare, Superdex200 16/600) equilibrated with 25 mM Tris pH 7.5, 150 mM NaCl, and 5 mM dithiothreitol. Finally, the eluted proteins were concentrated to 16 mg/mL and stored at −80 °C.

### Crystal Structure Determination

Crystals of HaloTag-BTTA complex were prepared as described previously.^15^ Crystals were harvested into cryo-solution containing 30% (v/v) glycerol and flash-frozen in liquid nitrogen. X-ray diffraction data were collected at the beamline 7A of the Pohang Accelerator Laboratory and processed by the HKL2000 program.^32^ The crystal structure was solved by the molecular replacement by Phenix^33^ using apo-HaloTag (PDB ID 5Y2X) as a search model. Model building and refinement were carried out using Coot^34^ and Phenix,^33^ respectively. Data collection and refinement statistics are summarized in Table S1. The coordinates and crystallographic structure factors of the HaloTag-BTTA complex have been deposited in the Protein Data Bank (PDB ID 8J1O).

### *In Vitro* GEN-Click Modification

Briefly, 2–4 mg/mL BSA was dissolved in Dulbecco’s phosphate-buffered saline (DPBS), and 200 μM BTTAT and 1 μM HRP were added. To start the modification reaction, 1 mM H_2_O_2_ was added for 1 min, followed by Trolox (final concentration of 10 mM) and sodium ascorbate (final concentration of 10 mM) to quench the peroxidase reaction. This solution was then passed through an Amicon filter (cutoff 10 kDa) and washed twice using DPBS to remove unreacted BTTAT. The concentrated BSA was then brought back to the original concentration of 2–4 mg/mL by adding CaCl_2_-free DPBS as needed. Next, 200 μM CuSO_4_ was incubated for 15–20 min, and an Amicon filter was used again to remove any unbound CuSO_4_ from the solution. After washing 3–4 times, the collected concentrate was measured for concentration and used as BSA-GEN-Click as required.

### General Transfection Protocol

For transfection, cells were cultured in Dulbecco’s modified eagle medium (DMEM) (Hyclone, SH30243) supplemented with 10% FBS, 2 mM l-glutamine, 50 units/mL penicillin, and 50 μg/mL streptomycin at 37 °C under 5% CO_2_, for a 12-well plate at 60–70% confluency; 1000 ng of plasmid DNA was mixed with 2 μg of polyethylenimine (PEI, Polysciences, 23966) using 100 μL of no-FBS and DMEM and added to the well. After 2–3 h of addition, media was changed to the full media described above, and transfected cells were used for imaging or labeling experiments after 20–24 h of transfection.

### General Imaging Protocol for GEN-Click Based Biotinylation

For imaging experiments, HEK-AD or HEK293T cells were cultured in DMEM supplemented with 10% FBS, 2 mM l-glutamine, 50 units/mL penicillin, and 50 μg/mL streptomycin at 37 °C under 5% CO_2_. After transfecting APEX2-TM or HRP-TM, cells were incubated overnight (12 h) with ManAz (4AcManAz, Click Chemistry Tools, Product No. 1084, 50 μM), AHA (100 μM), or alkyne-choline (propargyl-choline, synthesized using the reported protocol,^5^ 100 μM). After overnight incubation with azide- or alkyne-conjugated metabolic precursor molecules, cells were washed two times; 200 μM BTTAT was preincubated with 100 μM CuSO_4_ in CaCl_2_-free DPBS for 5–10 min and added to cultured cells. Immediately, 10 mM H_2_O_2_ was used to prepare 1 mM H_2_O_2_ in the reaction medium, thus creating the GEN-Click system (for the HaloTag-based approach, 10 μM BTTA-PEG-HTL was preincubated with CuSO_4_ and incubated and added to the cells for 30 min); cells were thoroughly washed three times using CaCl_2_-free DPBS and 100 μM desthiobiotin-alkyne along with 2 mM sodium ascorbate, followed by incubation for 10 min. Then, cells were washed with DPBS three times and cells were fixed with 4% paraformaldehyde solution (Chembio, CBPF-9004) in DPBS at room temperature for 15 min. Cells were then washed with DPBS two times and permeabilized with chilled methanol at −20 °C for 5 min. Cells were washed again two times with DPBS and blocked for 30 min with 2% BSA (Millipore, 82-100-6) in DPBS (“blocking buffer”) at room temperature. To detect APEX2 fusion protein expression, cells were incubated with mouse anti-V5 antibody (Invitrogen, cat. no. R960-25, 1:5000 dilution) for 1 h at room temperature. After washing four times with Tris-buffered saline with 0.1% Tween20 detergent (TBST) every 5 min, the cells were simultaneously incubated with secondary Alexa Fluor 568-goat antimouse IgG (Invitrogen, cat. no. A-11004, 1:1000 dilution) for 30 min at room temperature.

### General Western Blotting Protocol for GEN-Click-Based Biotinylation

For GEN-Click experiments, HEK-293T cells were cultured in DMEM supplemented with 10% FBS, 2 mM l-glutamine, 50 units/mL penicillin, and 50 μg/mL streptomycin at 37 °C under 5% CO_2_. After transfecting APEX2-TM or HRP-TM, cells were incubated overnight (12 h) with ManAz (50 μM), AHA (100 μM), or alkyne-choline (propargyl choline, 100 μM). After overnight incubation with azide- or alkyne-conjugated metabolic precursor molecules, cells were washed two times; 200 μM BTTAT was preincubated with 100 μM CuSO_4_ in CaCl_2_-free DPBS for 5–10 min and added to cultured cells. Immediately, 10 mM H_2_O_2_ was used to prepare 1 mM H_2_O_2_ in the reaction medium, thus creating the GEN-Click system; cells were thoroughly washed three times using CaCl_2_-free DPBS and 100 μM desthiobiotin-alkyne along with 2 mM sodium ascorbate followed by incubation for 10 min. Then, cells were washed with DPBS three times, and RIPA lysis buffer (Elpis biotech, EBA-1149) was added after removal of DPBS. Lysis was performed for 30 min at 4 °C. Then, the sample was loaded into 8% SDS-PAGE gel and run at 150 V for 60 min. After separation, proteins on the gel were transferred to the nitrocellulose membrane at 400 mA for 90 min. The protein loading level was checked via Ponceau staining, and Ponceau was removed by 1xTBST buffer. Blocking was performed with 2% skim milk in TBST for 1 h. The blocking solution was replaced with the primary antibody in 2% skim milk and incubated for 1 h. After washing with 1xTBST buffer four times (each for 5 min), the membrane was incubated with a secondary antibody or SA-HRP in 2% skim milk in TBST for 30 min. After washing with 1xTBST buffer four times, developing was done using an ECL kit (Biorad, 1705061) and images were captured using a Gel doc machine (Genesys).

### Protocol for Coculture-Based Metabolite Transfer

For metabolite-transferring experiments, HEK-293T cells were cultured in DMEM supplemented with 10% FBS, 2 mM l-glutamine, 50 units/mL penicillin, and 50 μg/mL streptomycin at 37 °C under 5% CO_2_. Metabolite recipient cells were transfected with APEX2-V5-TM and metabolite donor cells were incubated with 50 μM ManAz or 100 μM alkyne-choline for 24 h. After 24 h, cells were washed two times, incubated with Cell-Tracker Green for 30 min, and again washed two times with DMEM and trypsinized with four times the amount of trypsin used. DMEM was used to quench the trypsinization; the recipient and donor cells were mixed in a 1:1 ratio in a tube and centrifuged to remove trypsin-containing media. The pellet was dissolved in an appropriate amount of fresh medium, and cells were mixed gently using a pipet and left in the cell culture plate overnight. For the gap junction inhibitor treatment, carbenoxolone (CBX, 100 μM) was treated for 12 h at this coculture step. GEN-Click was carried out according to a previously described protocol.

## Supporting Information Available

Materials, experimental details, and related supporting data as described in the text (PDF)
Transparent Peer Review report available (PDF)

## References

[ref1] Tornøe C. W.; Christensen C.; Meldal M. Peptidotriazoles on Solid Phase: [1,2,3]-Triazoles by Regiospecific Copper(I)-Catalyzed 1,3-Dipolar Cycloadditions of Terminal Alkynes to Azides. J. Org. Chem. 2002, 67 (9), 3057–3064. 10.1021/jo011148j. 11975567

[ref2] Wang Q.; Chan T. R.; Hilgraf R.; Fokin V. V.; Sharpless K. B.; Finn M. G. Bioconjugation by Copper(I)-Catalyzed Azide-Alkyne [3 + 2] Cycloaddition. J. Am. Chem. Soc. 2003, 125 (11), 3192–3193. 10.1021/ja021381e. 12630856

[ref3] Kiick K. L.; Saxon E.; Tirrell D. A.; Bertozzi C. R. Incorporation of azides into recombinant proteins for chemoselective modification by the Staudinger ligation. Proc. Natl. Acad. Sci. U. S. A. 2002, 99 (1), 19–24. 10.1073/pnas.012583299. 11752401 PMC117506

[ref4] Saxon E.; Bertozzi C. R. Cell Surface Engineering by a Modified Staudinger Reaction. Science 2000, 287 (5460), 2007–2010. 10.1126/science.287.5460.2007. 10720325

[ref5] Li C.; Key J. A.; Jia F.; Dandapat A.; Hur S.; Cairo C. W. Practical labeling methodology for choline-derived lipids and applications in live cell fluorescence imaging. Photochem. Photobiol. 2014, 90 (3), 686–695. 10.1111/php.12234. 24383866

[ref6] Besanceney-Webler C.; Jiang H.; Zheng T.; Feng L.; Soriano del Amo D.; Wang W.; Klivansky L. M.; Marlow F. L.; Liu Y.; Wu P. Increasing the efficacy of bioorthogonal click reactions for bioconjugation: a comparative study. Angew. Chem., Int. Ed. 2011, 50 (35), 8051–8056. 10.1002/anie.201101817. PMC3465470 21761519

[ref7] Liu L.; Zhang D.; Johnson M.; Devaraj N. K. Light-activated tetrazines enable precision live-cell bioorthogonal chemistry. Nat. Chem. 2022, 14 (9), 1078–1085. 10.1038/s41557-022-00963-8. 35788560 PMC10198265

[ref8] Yang J.; Šečkutė J.; Cole C. M.; Devaraj N. K. Live-Cell Imaging of Cyclopropene Tags with Fluorogenic Tetrazine Cycloadditions. Angew. Chem., Int. Ed. 2012, 51 (30), 7476–7479. 10.1002/anie.201202122. PMC3431913 22696426

[ref9] Gordon C. K. L.; Wu D.; Pusuluri A.; Feagin T. A.; Csordas A. T.; Eisenstein M. S.; Hawker C. J.; Niu J.; Soh H. T. Click-Particle Display for Base-Modified Aptamer Discovery. ACS Chem. Biol. 2019, 14 (12), 2652–2662. 10.1021/acschembio.9b00587. 31532184 PMC6929039

[ref10] Hardy M. D.; Yang J.; Selimkhanov J.; Cole C. M.; Tsimring L. S.; Devaraj N. K. Self-reproducing catalyst drives repeated phospholipid synthesis and membrane growth. Proc. Natl. Acad. Sci. U. S. A. 2015, 112 (27), 8187–8192. 10.1073/pnas.1506704112. 26100914 PMC4500204

[ref11] Uttamapinant C.; Tangpeerachaikul A.; Grecian S.; Clarke S.; Singh U.; Slade P.; Gee K. R.; Ting A. Y. Fast, Cell-Compatible Click Chemistry with Copper-Chelating Azides for Biomolecular Labeling. Angew. Chem., Int. Ed. 2012, 51 (24), 5852–5856. 10.1002/anie.201108181. PMC3517120 22555882

[ref12] Chen J.; Wang J.; Li K.; Wang Y.; Gruebele M.; Ferguson A. L.; Zimmerman S. C. Polymeric “Clickase” Accelerates the Copper Click Reaction of Small Molecules, Proteins, and Cells. J. Am. Chem. Soc. 2019, 141 (24), 9693–9700. 10.1021/jacs.9b04181. 31124359 PMC13229381

[ref13] Li S.; Wang L.; Yu F.; Zhu Z.; Shobaki D.; Chen H.; Wang M.; Wang J.; Qin G.; Erasquin U. J.; et al. Copper-catalyzed click reaction on/in live cells. Chem. Sci. 2017, 8 (3), 2107–2114. 10.1039/C6SC02297A. 28348729 PMC5365239

[ref14] Los G. V.; Encell L. P.; McDougall M. G.; Hartzell D. D.; Karassina N.; Zimprich C.; Wood M. G.; Learish R.; Ohana R. F.; Urh M.; et al. HaloTag: A Novel Protein Labeling Technology for Cell Imaging and Protein Analysis. ACS Chem. Biol. 2008, 3 (6), 373–382. 10.1021/cb800025k. 18533659

[ref15] Mishra P. K.; Kang M.-G.; Lee H.; Kim S.; Choi S.; Sharma N.; Park C.-M.; Ko J.; Lee C.; Seo J. K.; et al. A chemical tool for blue light-inducible proximity photo-crosslinking in live cells. Chem. Sci. 2022, 13 (4), 955–966. 10.1039/D1SC04871F. 35211260 PMC8790779

[ref16] Frei M. S.; Tarnawski M.; Roberti M. J.; Koch B.; Hiblot J.; Johnsson K. Engineered HaloTag variants for fluorescence lifetime multiplexing. Nat. Methods 2022, 19 (1), 65–70. 10.1038/s41592-021-01341-x. 34916672 PMC8748199

[ref17] Lam S. S.; Martell J. D.; Kamer K. J.; Deerinck T. J.; Ellisman M. H.; Mootha V. K.; Ting A. Y. Directed evolution of APEX2 for electron microscopy and proximity labeling. Nat. Methods 2015, 12 (1), 51–54. 10.1038/nmeth.3179. 25419960 PMC4296904

[ref18] Chen C.-L.; Hu Y.; Udeshi N. D.; Lau T. Y.; Wirtz-Peitz F.; He L.; Ting A. Y.; Carr S. A.; Perrimon N. Proteomic mapping in live Drosophila tissues using an engineered ascorbate peroxidase. Proc. Natl. Acad. Sci. U. S. A. 2015, 112 (39), 12093–12098. 10.1073/pnas.1515623112. 26362788 PMC4593093

[ref19] Rhee H. W.; Zou P.; Udeshi N. D.; Martell J. D.; Mootha V. K.; Carr S. A.; Ting A. Y. Proteomic mapping of mitochondria in living cells via spatially restricted enzymatic tagging. Science 2013, 339 (6125), 1328–1331. 10.1126/science.1230593. 23371551 PMC3916822

[ref20] Martell J. D.; Deerinck T. J.; Sancak Y.; Poulos T. L.; Mootha V. K.; Sosinsky G. E.; Ellisman M. H.; Ting A. Y. Engineered ascorbate peroxidase as a genetically encoded reporter for electron microscopy. Nat. Biotechnol. 2012, 30 (11), 1143–1148. 10.1038/nbt.2375. 23086203 PMC3699407

[ref21] Lee S. Y.; Kang M. G.; Shin S.; Kwak C.; Kwon T.; Seo J. K.; Kim J. S.; Rhee H. W. Architecture Mapping of the Inner Mitochondrial Membrane Proteome by Chemical Tools in Live Cells. J. Am. Chem. Soc. 2017, 139 (10), 3651–3662. 10.1021/jacs.6b10418. 28156110

[ref22] Peters T.; Blumenstock F. A. Copper-binding Properties of Bovine Serum Albumin and Its Amino-terminal Peptide Fragment. J. Biol. Chem. 1967, 242 (7), 1574–1578. 10.1016/S0021-9258(18)96130-2. 6023222

[ref23] Agard N. J.; Prescher J. A.; Bertozzi C. R. A Strain-Promoted [3 + 2] Azide-Alkyne Cycloaddition for Covalent Modification of Biomolecules in Living Systems. J. Am. Chem. Soc. 2004, 126 (46), 15046–15047. 10.1021/ja044996f. 15547999

[ref24] Zhao N.; Kamijo K.; Fox P. D.; Oda H.; Morisaki T.; Sato Y.; Kimura H.; Stasevich T. J. A genetically encoded probe for imaging nascent and mature HA-tagged proteins in vivo. Nat. Commun. 2019, 10 (1), 2947 10.1038/s41467-019-10846-1. 31270320 PMC6610143

[ref25] Li Y.; Liu W.; Tang Q.; Fan X.; Hao Y.; Gao L.; Li Z.; Cheng B.; Chen X. Gap-Junction-Dependent Labeling of Nascent Proteins in Multicellular Networks. ACS Chem. Biol. 2019, 14 (2), 182–185. 10.1021/acschembio.8b01065. 30715839

[ref26] Choi C. R.; Rhee H. W. Proximity labeling: an enzymatic tool for spatial biology. Trends Biotechnol. 2022, 40 (2), 145–148. 10.1016/j.tibtech.2021.09.008. 34663510

[ref27] Sletten E. M.; Bertozzi C. R. Bioorthogonal chemistry: fishing for selectivity in a sea of functionality. Angew. Chem., Int. Ed. 2009, 48 (38), 6974–6998. 10.1002/anie.200900942. PMC2864149 19714693

[ref28] Wang N.; De Bock M.; Decrock E.; Bol M.; Gadicherla A.; Vinken M.; Rogiers V.; Bukauskas F. F.; Bultynck G.; Leybaert L. Paracrine signaling through plasma membrane hemichannels. Biochim. Biophys. Acta, Biomembr. 2013, 1828 (1), 35–50. 10.1016/j.bbamem.2012.07.002. PMC3666170 22796188

[ref29] Monterisi S.; Michl J.; Hulikova A.; Koth J.; Bridges E. M.; Hill A. E.; Abdullayeva G.; Bodmer W. F.; Swietach P. Solute exchange through gap junctions lessens the adverse effects of inactivating mutations in metabolite-handling genes. eLife 2022, 11, e78425 10.7554/eLife.78425. 36107487 PMC9534548

[ref30] Lee S.; Chung C. Y.-S.; Liu P.; Craciun L.; Nishikawa Y.; Bruemmer K. J.; Hamachi I.; Saijo K.; Miller E. W.; Chang C. J. Activity-Based Sensing with a Metal-Directed Acyl Imidazole Strategy Reveals Cell Type-Dependent Pools of Labile Brain Copper. J. Am. Chem. Soc. 2020, 142 (35), 14993–15003. 10.1021/jacs.0c05727. 32815370 PMC7877313

[ref31] Davis H. J.; Ward T. R. Artificial Metalloenzymes: Challenges and Opportunities. ACS Cent. Sci. 2019, 5 (7), 1120–1136. 10.1021/acscentsci.9b00397. 31404244 PMC6661864

[ref32] Otwinowski Z.; Minor W. [20] Processing of X-ray diffraction data collected in oscillation mode. Methods Enzymol. 1997, 276, 307–326. 10.1016/S0076-6879(97)76066-X. 27754618

[ref33] Adams P. D.; Afonine P. V.; Bunkoczi G.; Chen V. B.; Davis I. W.; Echols N.; Headd J. J.; Hung L.-W.; Kapral G. J.; Grosse-Kunstleve R. W.; et al. PHENIX: a comprehensive Python-based system for macromolecular structure solution. Acta Crystallogr., Sect. D: Biol. Crystallogr. 2010, 66 (2), 213–221. 10.1107/S0907444909052925. 20124702 PMC2815670

[ref34] Emsley P.; Lohkamp B.; Scott W. G.; Cowtan K. Features and development of Coot. Acta Crystallogr., Sect. D: Biol. Crystallogr. 2010, 66, 486–501. 10.1107/S0907444910007493. 20383002 PMC2852313

