# Supplementary material for: GEN-Click: Genetically Encodable Click Reactions for Spatially Restricted Metabolite Labeling
Source: ACS Cent Sci. 2023 Jul 25;9(8):1650–7. doi: 10.1021/acscentsci.3c00511 (PMC10450880; doi:10.1021/acscentsci.3c00511)
Supplement: Supplementary file 1 — oc3c00511_si_001.pdf [file oc3c00511_si_001.pdf]

Supporting Information

GEN-Click: Genetically Encodable Click Reactions for Spatially Restricted Metabolite Labeling

Pratyush Kumar Mishra[1,*], Nirmali Sharma[1], Hyunwoo Kim[2], Changwook Lee[2], and Hyun-Woo Rhee[1,3,*]

[1]Department of Chemistry, Seoul National University, Seoul 08826, Korea

[2]Department of Biological Sciences, Ulsan National institute of Science and Technology, Ulsan 44919, Korea

[3]School of Biological Sciences, Seoul National University, Seoul 08826, Korea

*Corresponding authors (H.W.R., rheehw@snu.ac.kr ; P.K.M., pkmishra@snu.ac.kr)

Table of contents:

Table. S1……………………………………………………………………………………S3

Table. S2……………………………………………………………………………………S4

Figure S1……………………………………………………………………………………S5

Figure S2……………………………………………………………………………………S6

Figure S3……………………………………………………………………………………S7

Figure S4……………………………………………………………………………………S8

Figure S5……………………………………………………………………………………S9

Figure S6…………………………………………………………………………………… S10

Figure S7……………………………………………………………………………………S11

Figure S8…………………………………………………………………………………… S13

Scheme S1…………………………………………………………………………………S15

Synthesis Protocol…………………………………………………………………………S16

Table 1. Data collection and refinement statistics.

	HaloTag-BTTA
Dataset	Native
X-ray source	Beamline 7A, PAL
Temperature (K)	100
Space group:	$P4_32_12$
Cell parameters	
a, b, c (Å)	63.090, 63.090, 163.680
α, β, γ (°)	90.000, 90.000, 90.000
Data processing	
Wavelength (Å)	0.97960
Resolution (Å)	50.00–1.99
CC1/2	0.999 (0.906)
I/σ	22.03 (4.25)
Completeness (%)	99.8 (99.9)
Redundancy	4.5 (3.8)
Measured reflections	192,853
Unique reflections	23,615
Refinement statistics	
Resolution (Å)	30.16–1.99
Reflections	23,615
Number of atoms	
Protein	4008
Water	191
R-factor (%)	15.53
R_{free} (%)	19.81
RMSD	
Bond lengths (Å)	0.012
Bond angles (°)	0.963
Ramachandran plot, residues in	
Favored regions (%)	95.89
Allowed regions (%)	4.11
Disallowed regions (%)	0.00

*Highest resolution shell is shown in parenthesis.

The coordinates and crystallographic structure factors of the HaloTag-BTTA complex have been deposited in the Protein Data Bank (PDB ID: 8J1O).

Table S2. Construct Table

Name	Features	Promotor/ Vector	Details	Note
V5-APEX2-TM	*NotI*-Igk-V5-APEX2-*NheI*-TM-*XhoI*	CMV/ pCDNA5	**V5**: GKPIPNPLLGLDST<br>**Igk**:METDTLLLWVLLLWVPGSTGD<br>**TM**:AVGQDTQEVIVVPHSLPFKVVVISAILALVVLTIISLIILIMLWQKKPR	V5-APEX2-TM was used in PMID: 36265183
HRP-myc-TM	Igk-HA-*ApaI*-HRP-*SacII*-myc-TM- *NotI*	CMV/ pDisplay	**Igk**:METDTLLLWVLLLWVPGSTGD<br>**HA**: YPYDVPDYA<br>**myc**: EQKLISEEDL<br>**TM**:AVGQDTQEVIVVPHSLPFKVVVISAILALVVLTIISLIILIMLWQKKPR	-This plasmid was obtained from addgene (Plasmid# 44441).
HA-mCherry-TM	Igk-*ApaI*-Short OpsinTag-2X-HA-mCherry-TM-*NotI*	CMV/ pDisplay	**Igk**:METDTLLLWVLLLWVPGSTGD<br>**Short Opsin Tag:** GPNFYVPFSNKTG<br>**HA**: YPYDVPDYA<br>**TM**:AVGQDTQEVIVVPHSLPFKVVVISAILALVVLTIISLIILIMLWQKKPR	-Used as Receptor Protein for Frankenbody-APEX2<br>-Frankenbody sequence was obtained from addgene (Plasmid#129593).<br>-Short Opsin Tag was used as glycosylation motif.
Frankenbody-Alfa-APEX2	Igk-*ApaI*-*BglII*-15F11anti-HA scFV (Frankenbody)-AflII-Alfa-APEX2-Stop-*NotI*	CMV/ pDisplay	**Igk**:METDTLLLWVLLLWVPGSTGD<br>**Alfa**: SRLEEELRRRLTE	-Used as Ligand Protein for HA-mCherry-TM<br>-Alfa can be detected with GFP-conjugated Nanobody ALFA (i.e. anti-ALFA-GFP) (PMID: 32066961)

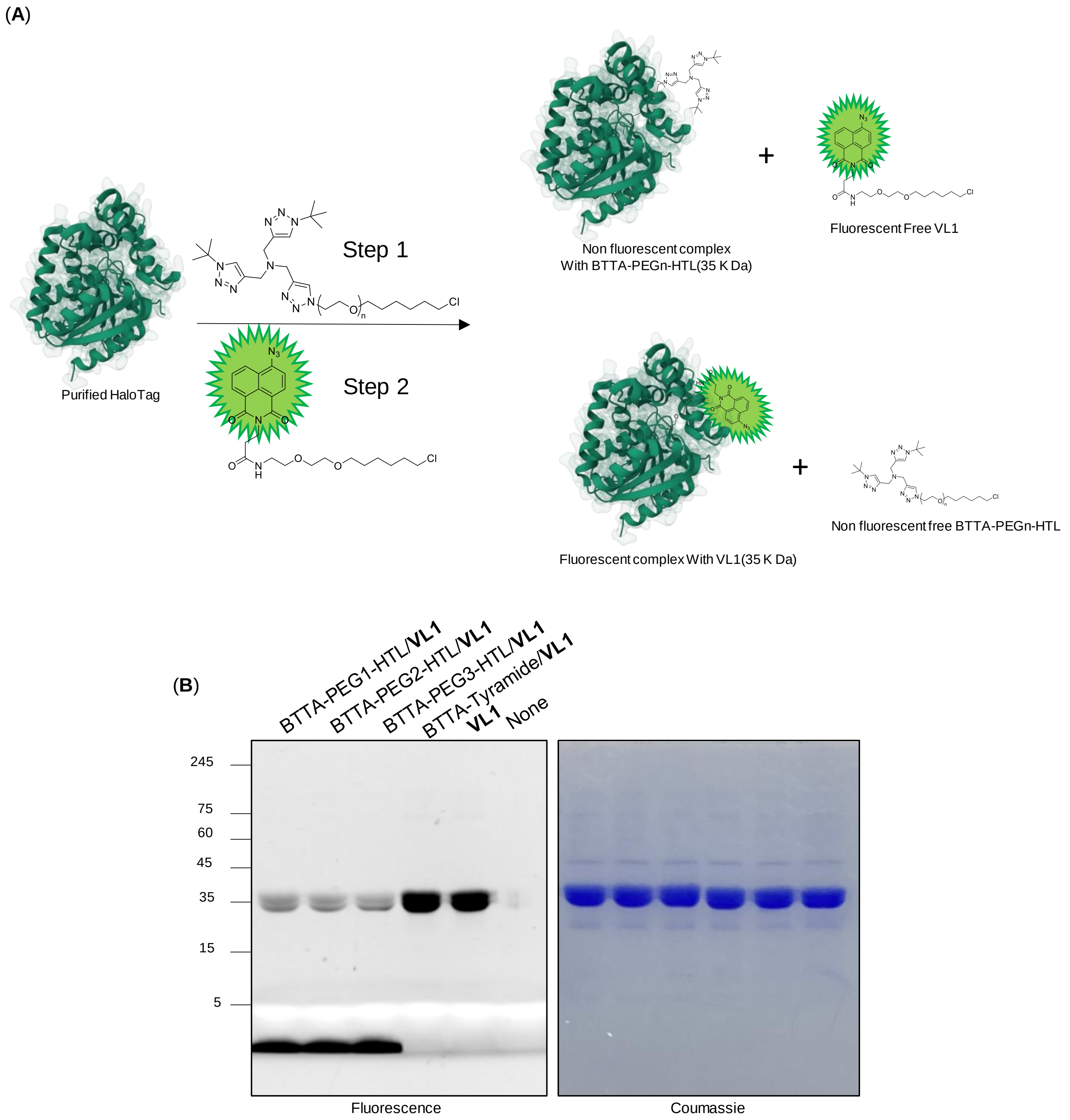

Figure S1 (Related to Figure 1). (**A**) Schematic representation of competitive HaloTag binding evaluation using fluorescent competitor ligand (VL1) (**B**) Fluorescence gel-imaging result showing HaloTag binding affinity of BTTA-PEGn-HTL ligands. BTTA-Tyramide (BTTAT) was used as a control.

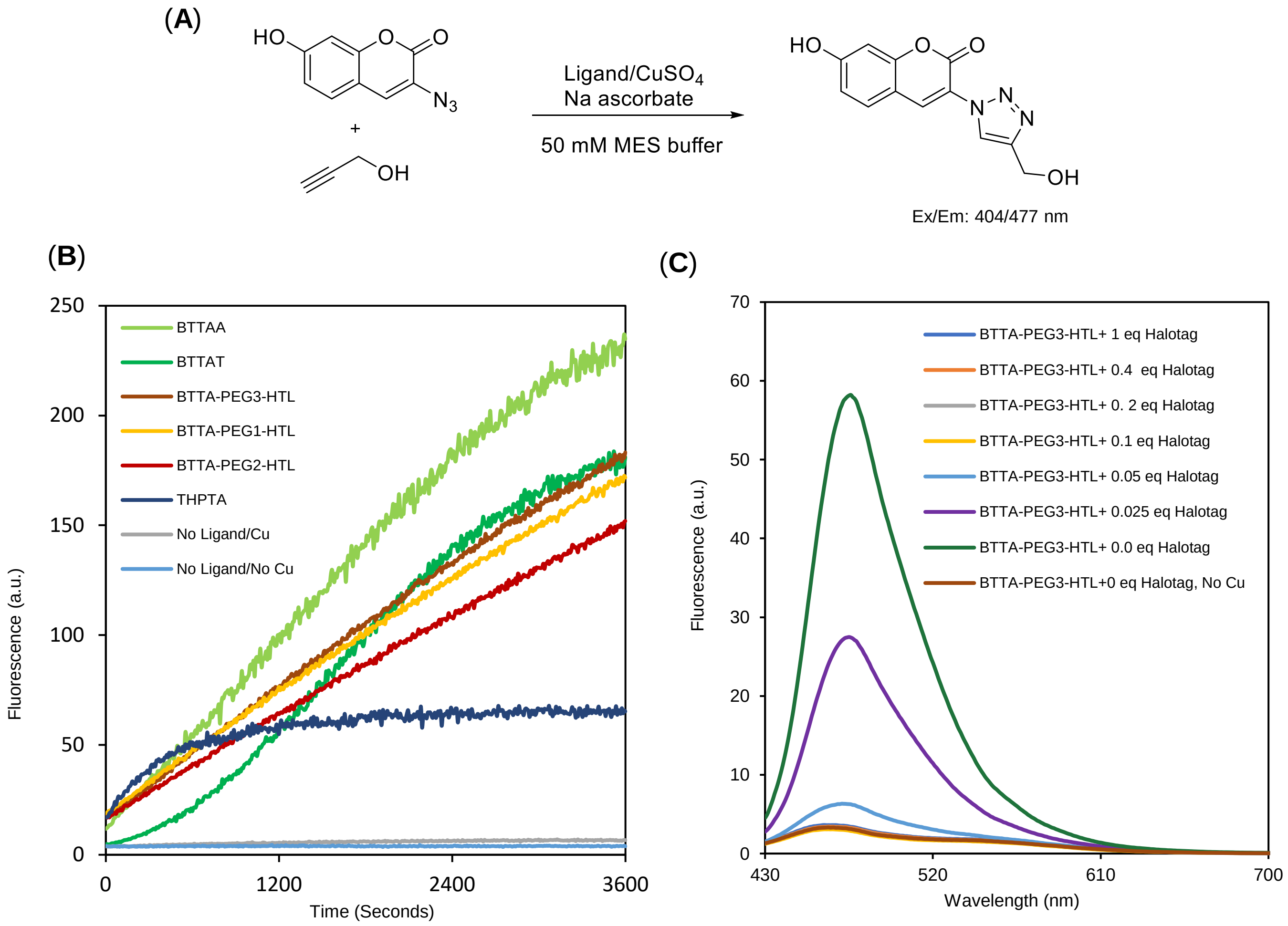

Figure S2. In vitro copper-click reaction monitoring using various copper-binding catalysts (**A**) Scheme of fluorescence turn on click reaction adduct formation using azidocoumarin and propargyl alcohol. (**B**) In-vitro click reaction monitoring result using BTTA-PEGn-HTL, THPTA and BTTAA using 10 µM $CuSO_4$ 50 µM ligand, 50 µM azidocoumarin, 100 µM propargyl alcohol and 2.5 mM sodium ascorbate. (**C**) Fluorescence spectra showing catalytic quenching effect of HaloTag protein (0.025 Eq. to 1 Eq.) when added to the reaction mixture of BTTA-PEG3-HTL (20 µM) containing 10 µM $CuSO_4$, 50 µM azidocoumarin, 100 µM propargyl alcohol and 2.5 mM sodium ascorbate.

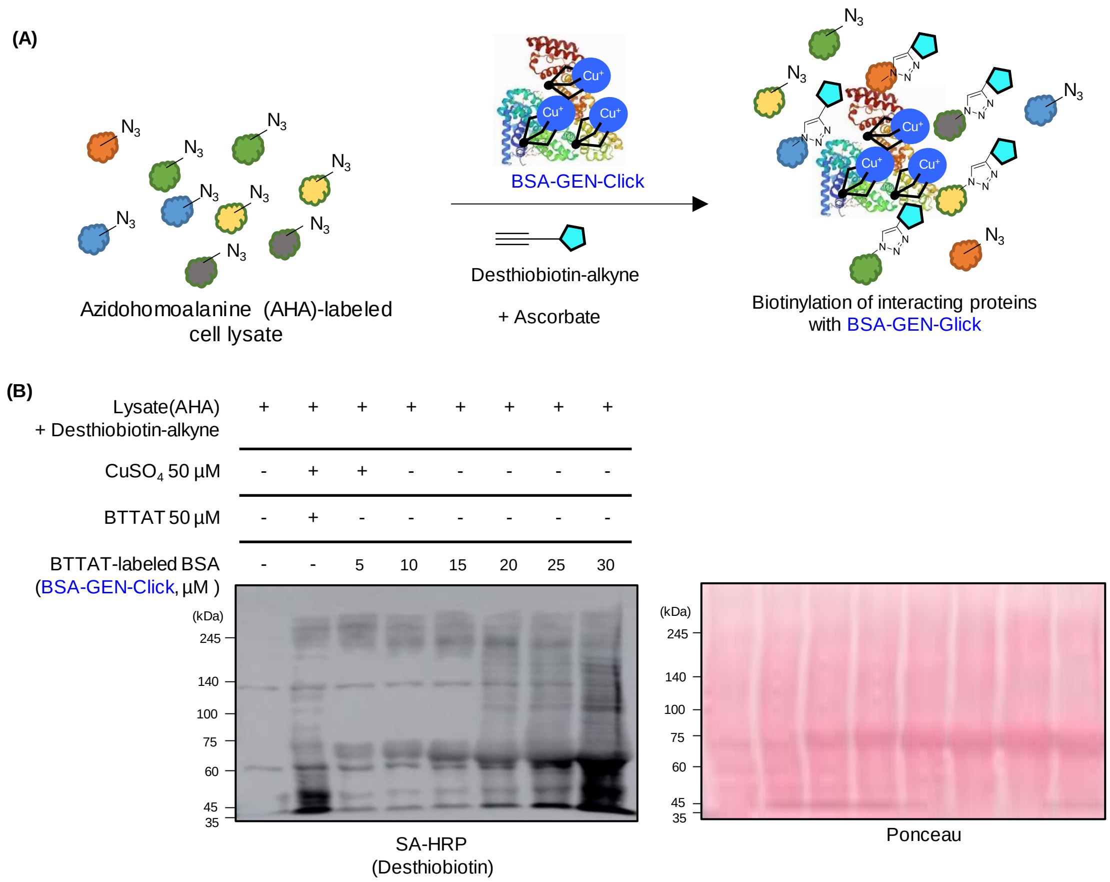

Figure S3. (A) Schematic representation of GEN-Click modification of azidohomoalanine (AHA) labeled proteins with Desthiobiotin-Alkyne and Cu-BTTAT modified BSA (BSA-GEN-Click). **(B)** Western blot analysis of BSA-GEN-Click (Cu/BTTAT-modified BSA) reaction on AHA-incorporated HEK293T cell lysate. 50 µM desthiobiotin-alkyne, 2.5 mM sodium ascorbate, and different concentrations of BSA-GEN-Click (5-30 µM) or free BTTAT (50 µM) were incubated with the lysate for 30 min at room temperature. Streptavidin-HRP (SA-HRP) was used for the western blot analysis of desthiobiotin modified proteins, which were the products of CuAAC reactions by BSA-GEN-Click.

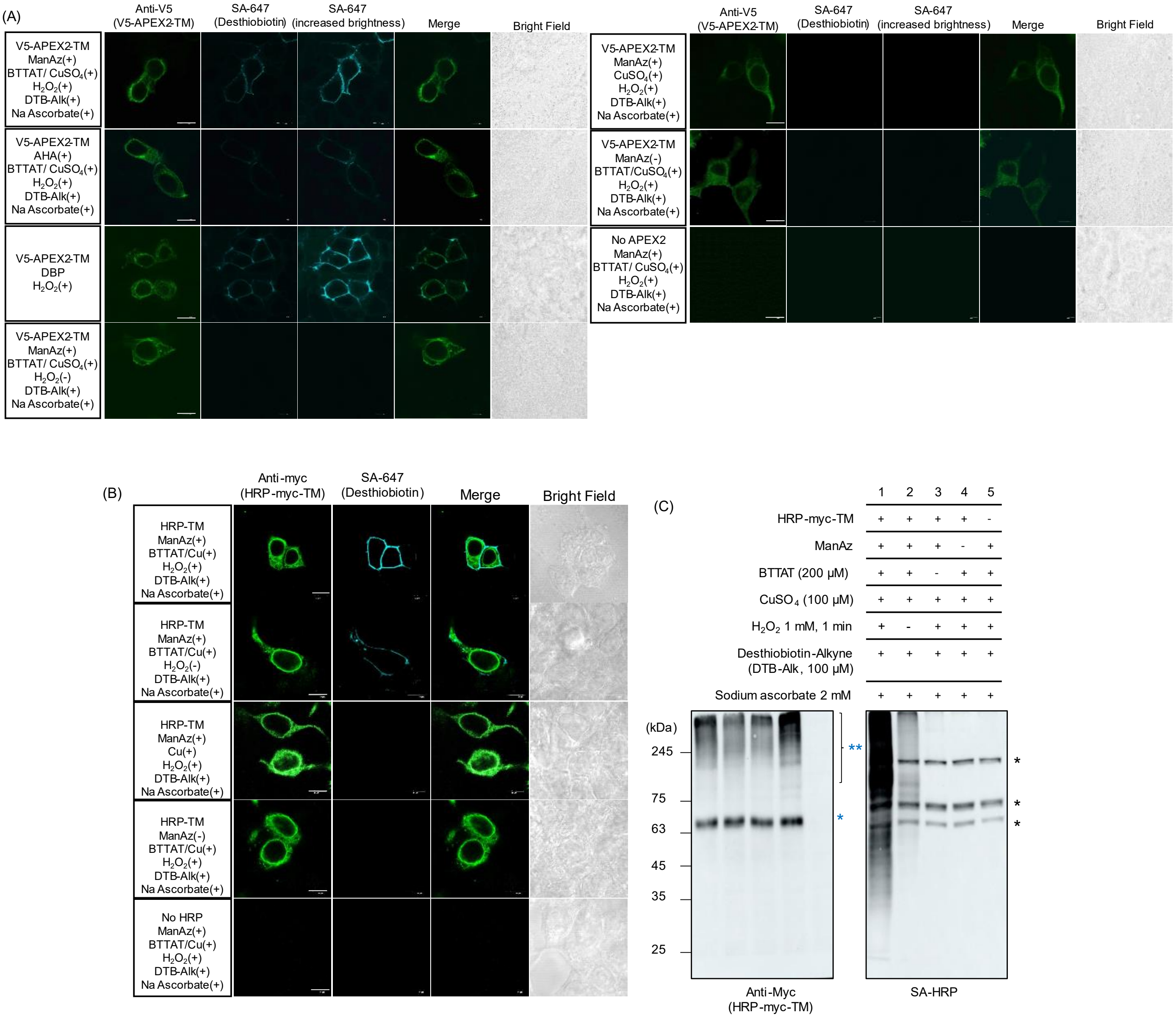

Figure S4 (Related to Figure 3). Cell surface proteome labeling using GEN-Click. (**A**) Confocal imaging results of GEN-Click biotinylation using mannose azide (ManAz) or azidohomoalanine (AHA). For GEN-Clic k labeling, V5-APEX2-TM expressing cells were treated with ManAz (50 μM) or azidohomoalanine (100 μ M) for overnight (12 hrs) and Cu-BTTAT (100 μM, 1:2) were treated for GEN-Click labeling. Desthiobiotin-Alkyne (DTB-Alk, 100 μM) with 2 mM sodium ascorbate was used to perform click reaction. Expression le vels of V5-APEX2-TM were confirmed by anti-V5 and SA-647 was used to visualize desthiobiotin labeling. Scale Bar: 10 μm. In this experiment, Omission of BTTAT, ManAz or APEX-TM expression was used as c ontrols in this reaction. Desthiobiotin-phenol (DBP) labeling was conducted for the comparison. **(B)** Confocal microscope imaging results of GEN-Click biotiylation by using HRP-myc-TM expressing cells. HRP-myc-TM expressing cells were treated with ManAz (50 μM) for overnight (12 hrs) and Cu-BTTAT (100 μM, 1:2) we re treated for GEN-Click labeling. Desthiobiotin-Alkyne (DTB-Alk, 100 μM) with 2 mM sodiumascorbate wa s used to perform click reaction. Expression levels of HRP-myc-TM were confirmed by anti-Myc and SA-64 7 was used to visualize desthiobiotin labeling Scale Bar: 10 μm. (C) Western blot result of GEN-Click bioti nylation using HRP-myc-TM cells. Endogenous biotinylated proteins are marked with black asterisks in SA-H RP western blot and blue asterisk marks HRP-myc-TM in anti-Myc western blot. Double blue asterisks indic ate crosslinked HRP-myc-TM.

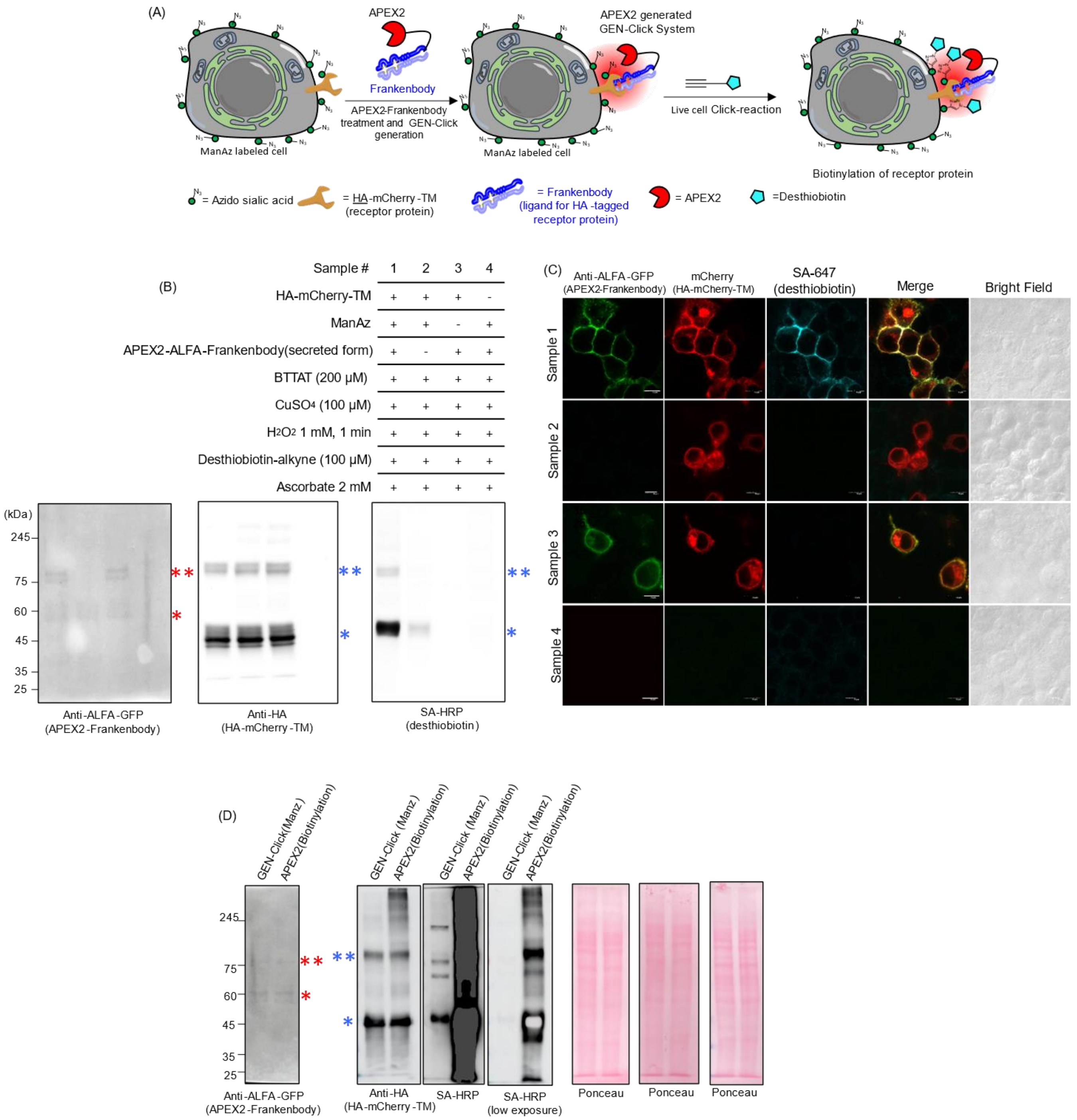

Figure S5. Biotinylation of receptor protein using ligand directed GEN-Click labeling. (A) Schematic representation of ligand-receptor mapping strategy using GEN-Click. (**B**) Western blot analysis results of GEN-click biotinylation of receptor protein (HA-mCherry-TM) by APEX2-ligand protein (APEX2-Alfa-Frankenbody). Receptor protein (HA-mCherry-TM) expressing cells were treated with ManAz (50 μM) for overnight. After the treatment of ligand protein (APEX2-Alfa-Frankenbody) on the receptor expression cells, Cu-BTTAT/H_2O_2 was treated for GEN-Click reaction. After this step, desthiobiotin-alkyne was used to perform click reaction. APEX2-Alfa-Frankenbody was collected from ligand secreting cells (No ManAz). see "General Western Blotting Protocol for GEN-Click Based Biotinylation" under Materials & Methods section for detail information. (**C**) Confocal imaging results of sample #1-4 in the experiment shown in (**B**). Scale Bar : 10 μm. (**D**) Western blot analysis results of HA-mCherry-TM expressing cells. ManAz (100 μM overnight incubation) was used as azide-incorporating block, APEX2-Alfa-Frankenbody was used as ligand on cell surface, and Desthiobiotin-alkyne was used to perform click reaction. For APEX2's phenoxyl radical labeling, 250 μM Desthiobiotin-Phenol and 1 mM H_2O_2 were incubated for 1 min and washed thrice with 5 mM NaN_3, 10 mM sodium ascorbate, and 10 mM Trolox. After the labeling, cells were lysed with RIPA lysis buffer. In the western blot results, red asterisk marks the ligand protein and blue asterisk marks receptor proteins. Double asterisks mark crosslinked complex in presence of H_2O_2.

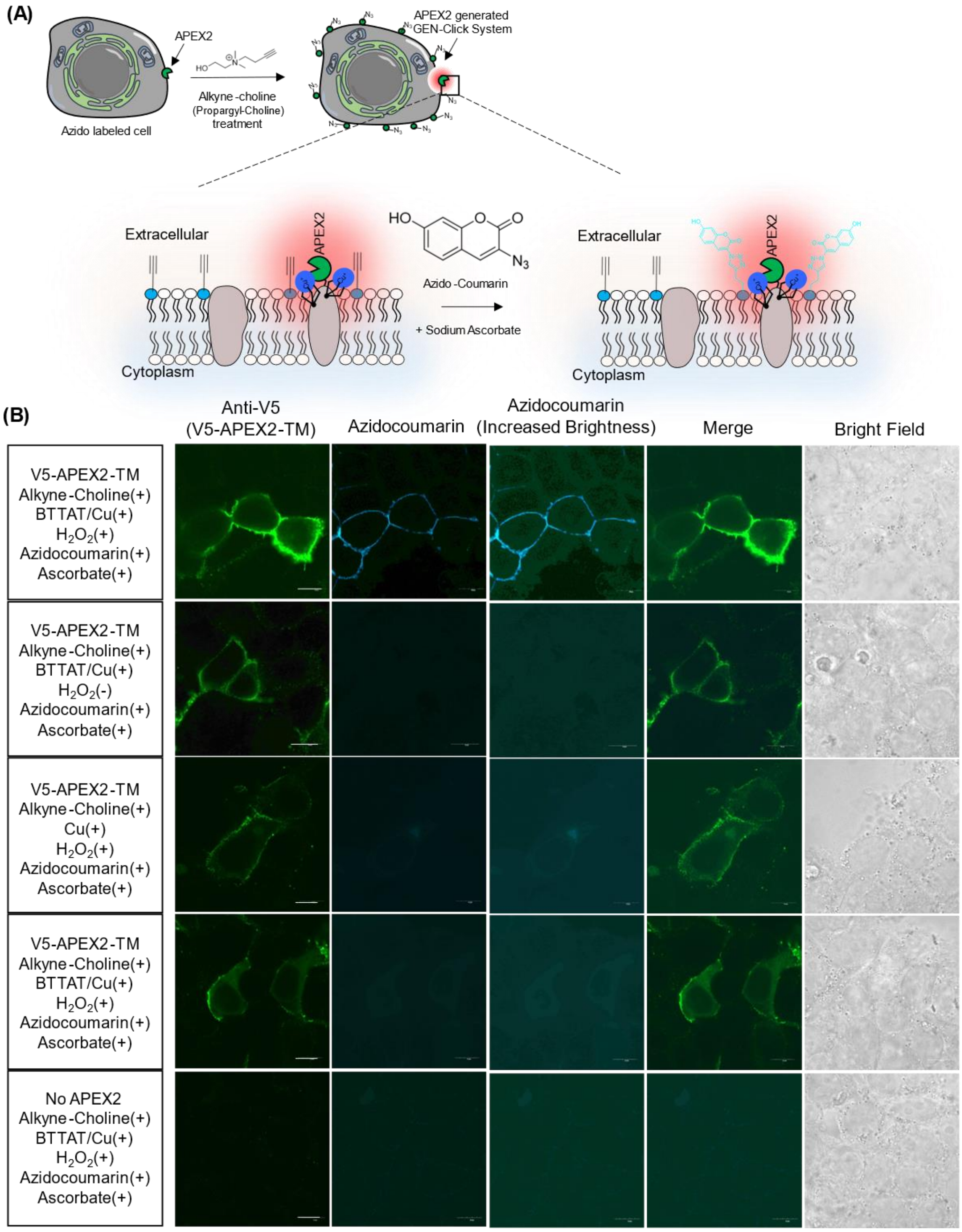

Figure S6. Cell surface phospholipid labeling using GEN-Click (**A**) Schematic representation of GEN-Click labeling on the metabolic incorporated alkyne-choline (propargyl choline) at the cell surface. APEX2-TM introduces GEN-Click catalyst (Cu-BTTAT) at the cell surface and alkyne choline-incorporated phospholipid can be labeled with azidocoumarin by GEN-Click at the cell surface. (**B**) Confocal microscope imaging results of phospholipid labeling by GEN-Click. Alkyne-choline (propargyl choline, 100 µM) was treated on the V5-APEX2-TM expressing cells for overnight. After overnight incubation, Cu-BTTAT (100 µM, 1:2) was treated for GEN-Click labeling. After this step, azidocoumarin (100 µM) with 2 mM sodium ascorbate was treated to initiate click reaction. Expression levels of V5-APEX2-TM were measured by anti-V5 and Ex=404/Em 477 was used to visualize clicked azidocoumarin labeling, Scale Bar: 10 µm.

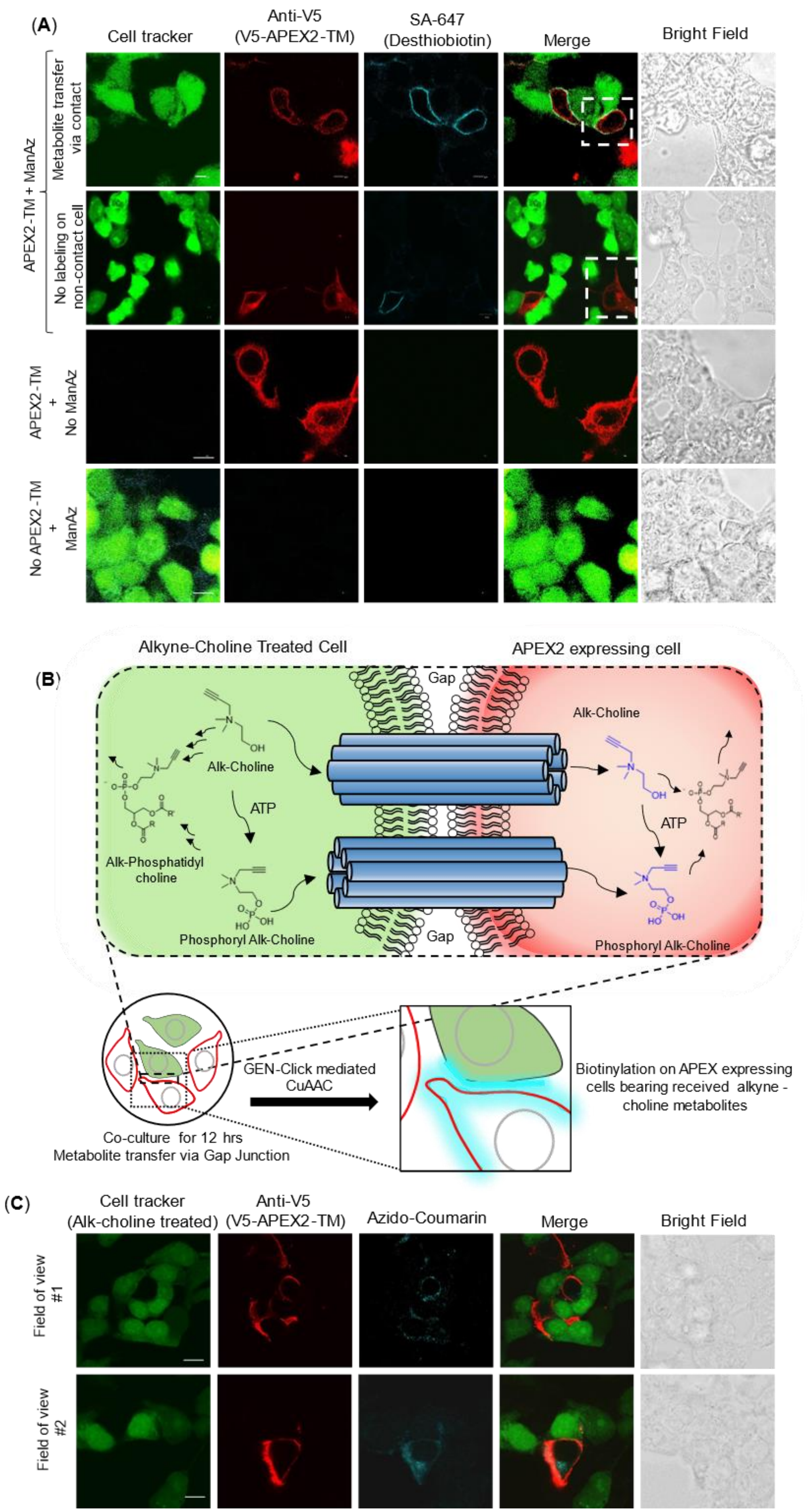

(A)
Cell tracker
Anti-V5 (V5-APEX2-TM)
SA-647 (Desthiobiotin)
Merge
Bright Field
APEX2-TM + ManAz
Metabolite transfer via contact
No labeling on non-contact cell
APEX2-TM + No ManAz
No APEX2-TM + ManAz
(B)
Alkyne-Choline Treated Cell
APEX2 expressing cell
Gap
Alk-Choline
ATP
Alk-Phosphatidyl choline
Phosphoryl Alk-Choline
Co-culture for 12 hrs
Metabolite transfer via Gap Junction
GEN-Click mediated CuAAC
Biotinylation on APEX expressing cells bearing received alkyne - choline metabolites
(C)
Cell tracker (Alk-choline treated)
Anti-V5 (V5-APEX2-TM)
Azido-Coumarin
Merge
Bright Field
Field of view #1
Field of view #2

Figure S7 (Related to Figure 4). Fluorescent visualization of mannose azide and alkyne-choline transfer at the cell-cell interface using GEN-Click (**A**) Additional fluorescence imaging results of intercellular mannose azide (ManAz) transfer with GEN-Click labeling in the co-cultured system of ManAz-treated cells (marked with Cell Tracker, green fluorescence) and V5-APEX2-V5 expressing cells (marked anti-V5/mouse-AF568 antibody). Desthiobiotin-labeled region is marked with Streptavidin-AF647 (SA-647). Scale bar: 10 μm. (**B**) Scheme of fluorescence imaging of alkyne-choline transferring event at the cell-cell contact site using GEN-Click (**C**) Confocal images of Alkyne-Choline metabolites being transferred to adjacent contact cells in a co-cultured condition. Expression levels of APEX2-TM confirmed by anti-V5/mouse-AF568 antibody. BTTAT-Cu labeled Alkyne-Choline visualized with copper-clicked azidocoumarin (Ex=404/Em 477). Scale Bar 10 μm.

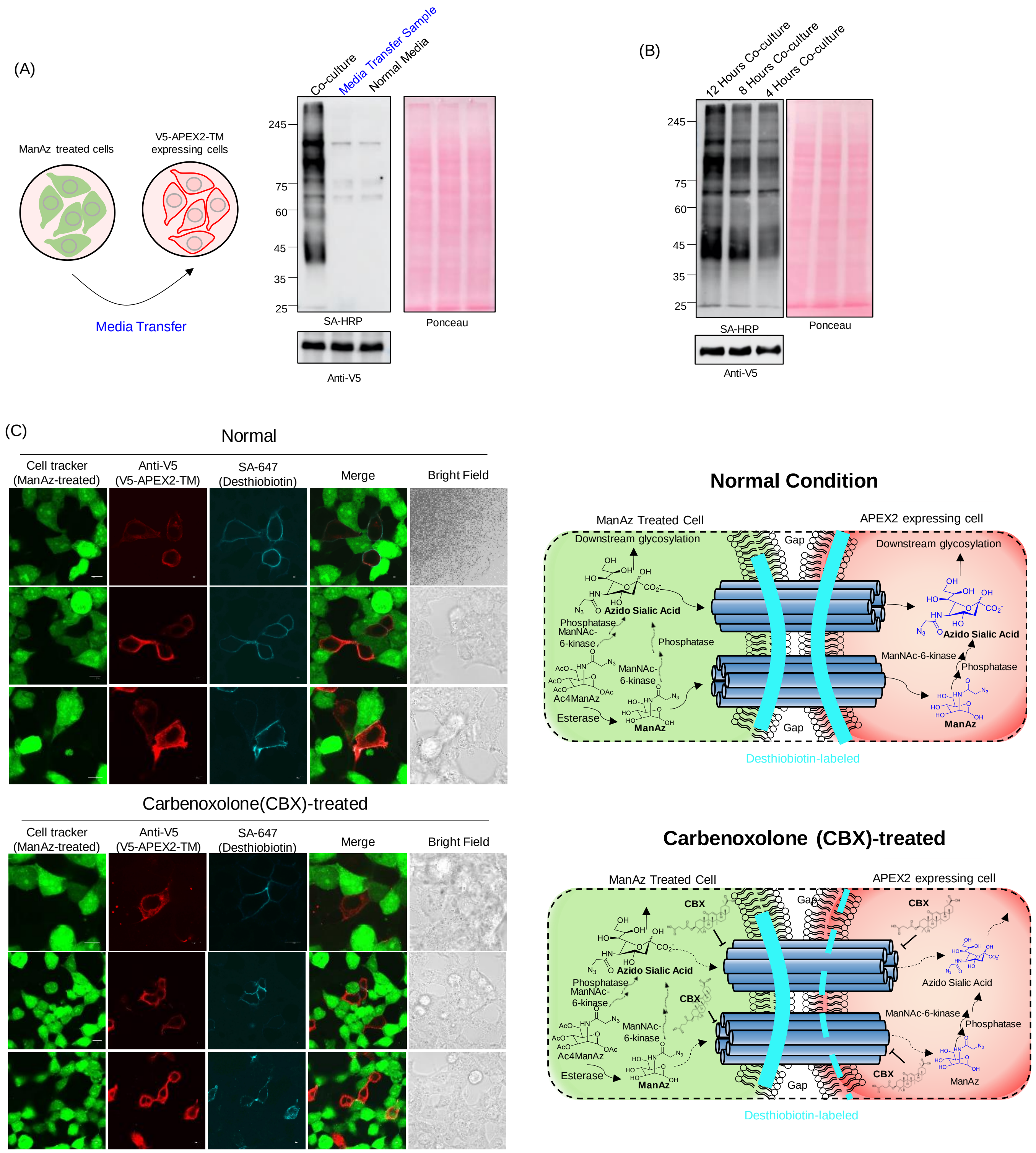

Figure S8 (related to Figure 4). GEN-Click assisted visualization of metabolite transfer events via gap junction (**A**) (Left) Scheme of media transfer experiment (lane 2 of B). (Right) Western blot analysis of GEN-Click biotinylated proteins in three different samples. Lane 1: GEN-Click labeled sample from normal co-cultured APEX2-V5-TM expressing cells and ManAz-treated cells Lane 2: GEN-Click labeled sample of APEX2-V5-TM expressing cells which were treated with incubated media of ManAz-labeled cells for overnight. Lane 3: GEN-Click labeled sample of APEX2-V5-TM expressing cells grown in normal media (DMEM). (**B**) Western blot analysis of GEN-Click biotinylated proteins in three different co-culture samples. In these samples, co-culture was done in a time dependent manner for 12, 8 and 4 hours respectively and GEN-Click system was applied to visualize transferred metabolites via biotinylation on azido-sialic acid incorporated glycan. In the results of (a) and (b), Streptavidin-HRP showed desthiobiotin-alkyne labeled

proteins and anti-V5 showed the expression level of V5-APEX2-TM. (**C**) (Left) Confocal images of gap junction inhibitor treated sample. For CBX-treated samples, CBX (100 µM) were treated for 12 hrs on the co-cultured of ManAz-treated cells (marked with Cell Tracker, green fluorescence) and V5-APEX2-TM expressing cells (marked with anti-V5/Mouse-568 antibody). Desthiobiotin-labeled molecules were visualized with Streptavidin-AF647 (SA-647). Control cells were cultured with normal media for 12 h. Both cells were treated with BTTAT/Cu and desthiobiotin-alkyne as described in the Material and Methods section of the manuscript. Scale bar 10 µm. (Right) Schematic representation of inhibition of ManAz transfer to adjacent cells by CBX. Transferred metabolites were marked with blue color.

Synthesis of Cu binding ligands

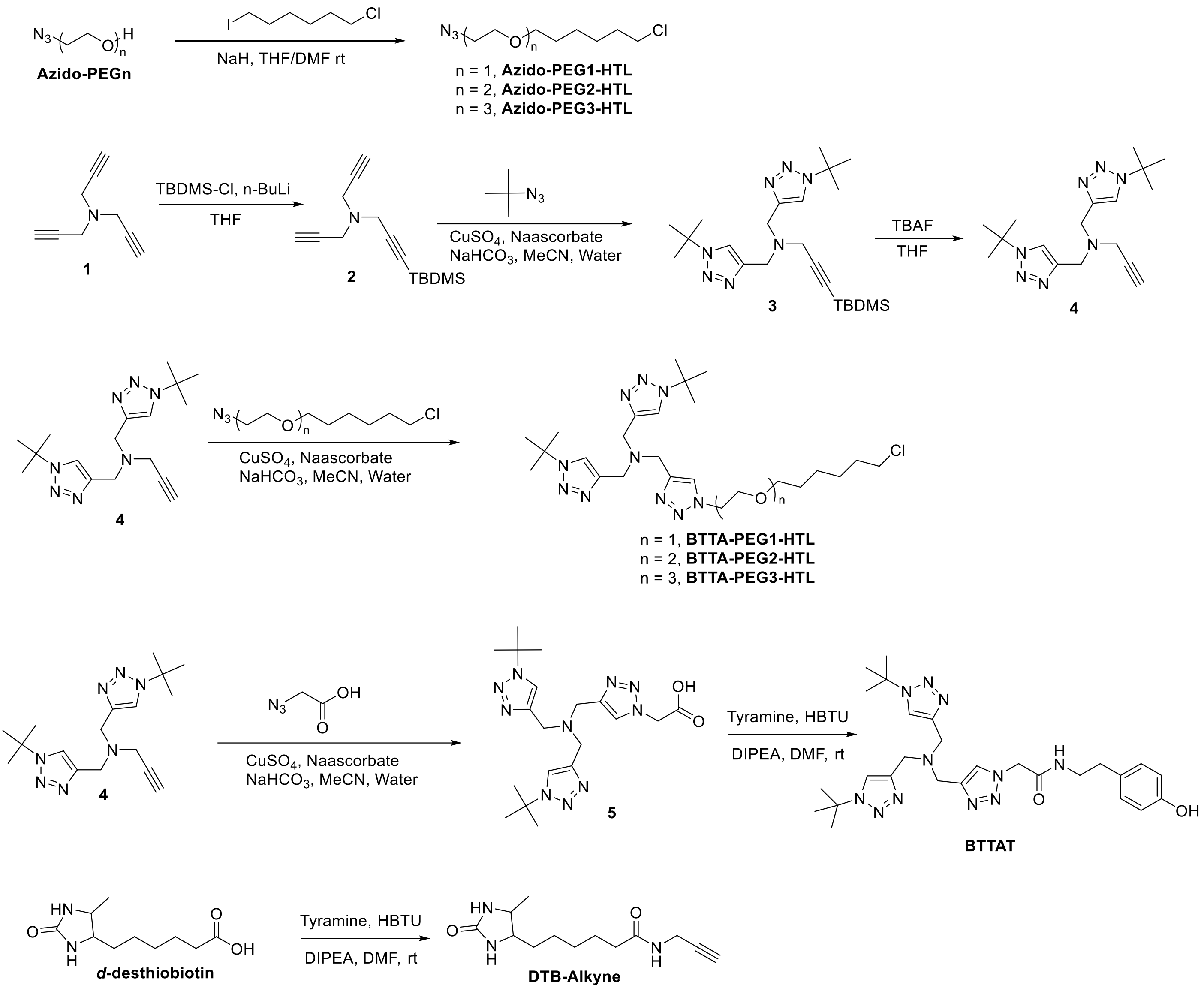

Scheme S1. Synthetic Scheme of **BTTA-PEGn-HTL** and **BTTAT**.

Synthesis protocol

General Synthesis of Azido-PEGn-HTL: 250 mg of Azido-PEGn was added to the flask containing 1.5 eq. of Sodium Hydride in 4 ml anhydrous THF:DMF(1:1) and mixture was stirred for 20-30 min, subsequently 1.5 Eq of 1-chloro-6-iodohexane was added dropwise and reaction mixture was stirred at room temperature for 12-14 hours, the mixture was concentrated under reduced pressure. The organic layer obtained by extraction with ethyl acetate/water was passed through a Na_2SO_4 Pad to remove remaining moisture, and then the solvent was removed under reduced pressure. Product was isolated using Silica-gel column chromatography (2-5% ethyl-acetate / n-hexane) to obtain Azido-PEGn-HTL as colourless oil.

Azido-PEG1-HTL: ^{1}H NMR (500 MHz, $CDCl_3$) δ 3.60 (t, *J* = 4.7 Hz, 2H), 3.53 (t, *J* = 6.7 Hz, 2H), 3.47 (t, *J* = 6.4 Hz, 2H), 3.36 (t, *J* = 4.8 Hz, 2H), 1.81 – 1.73 (m, 2H), 1.60 (dd, *J* = 14.0, 6.9 Hz, 2H), 1.51 – 1.34 (m, 4H). ^{13}C NMR (125 MHz, $CDCl_3$) δ 71.31 (s), 69.70 (s), 50.90 (s), 45.18 (s), 32.66 (s), 29.61 (s), 26.78 (s), 25.46 (s). MS (ESI+) m/z 180.11 $[M-N_2+H]^+$.

Azido-PEG2-HTL: Obtained data was matched with the literature data[1]. ^{1}H NMR (400 MHz, $CDCl_3$) δ 3.70 – 3.63 (m, 4H), 3.59 (dd, *J* = 6.0, 3.5 Hz, 2H), 3.53 (t, *J* = 6.7 Hz, 2H), 3.47 (t, *J* = 6.6 Hz, 2H), 3.39 (t, *J* = 5.1 Hz, 2H), 1.82 – 1.73 (m, 2H), 1.61 (dd, *J* = 14.2, 7.1 Hz, 2H), 1.49-1.35 (m, 4H).

Azido-PEG3-HTL: Obtained data was matched with the literature data[2]. ^{1}H NMR (400 MHz, $CDCl_3$) □ = 3.70-3.62 (m, 8H), 3.61-3.56 (m, 2H), 3.53 (t, *J* = 6.8 Hz, 2H), 3.46 (t, *J* = 6.8 Hz, 2H), 3.39 (t, *J* = 5.6 Hz, 2H), 1.82-1.73 (m, 2H), 1.64-1.55 (m, 2H), 1.50-1.32 (m, 4H).

3-(tert-butyldimethylsilyl)-N,N-di(prop-2-yn-1-yl)prop-2-yn-1-amine (2): Tripropargylamine (1.0 g, 7.6 mmol) was dissolved in 25 ml anhydrous tetrahydrofuran at -78 °C then 2.5 M of n-Butyllithium in n-hexane (1.84 ml, 4.6 mmol) was slowly added, and after stirring for 1 hour, a solution of t-Butyldimethylsilyl chloride (575 mg, 3.8 mmol) dissolved in 2.5 ml anhydrous tetrahydrofuran was added dropwise. After 30 minutes, the temperature was raised to room temperature and stirred for 18 hours. After the completion of reaction, the reaction was terminated by adding aqueous NH_4Cl solution, the mixture was concentrated under reduced pressure. The organic layer obtained by extraction with ethyl acetate/water was passed through a Na_2SO_4 Pad to remove remaining moisture, and then the solvent was removed under reduced pressure. Product was isolated using Silica-gel column chromatography (5% ethyl-acetate / n-hexane) to obtain 552 mg (59%) of (**2**) as white solid. ^{1}H NMR (400 MHz, $CDCl_3$) *δ* 3.51 (s, 2H), 3.48 (d, *J* = 2.4 Hz, 4H), 2.25 (t, *J* = 2.4 Hz, 2H), 0.94 (s, 9H), 0.10 (s, 6H); ^{13}C NMR (100 MHz, $CDCl_3$) *δ* 100.9, 100.6, 90.0, 88.4, 78.6, 73.2, 42.9, 41.7, 26.0, 16.5, 0.1, -4.6; MS (ESI+) m/z 246.3 $[M+H]^+$.

N,N-bis((1-(tert-butyl)-1H-1,2,3-triazol-4-yl)methyl)-3-(tert-butyldimethylsilyl)prop-2-yn-1-amine (3): Compound **(2)** (490 mg, 2.0 mmol) and tert-butyl azide (990 mg, 10 mmol) was taken in acetonitrile (28 mL), then $NaHCO_3$ (420 mg, 5.0 mmol), Sodium ascorbate (99 mg, 0.5 mmol), and $CuSO_4.5H_2O$ (125 mg, 0.5 mmol) were dissolved in water (14 mL) and added to the reaction mixture and stirred at room temperature for 18 hours. The organic layer obtained by extraction with ethyl acetate/water was passed through a Na2SO4 Pad to remove remaining moisture, and then the solvent was removed under reduced pressure. Product was isolated using Silica-gel column chromatography (50% ethyl acetate / n-hexane) to obtain 720 mg (81%) of (**3**) as white solid. ^{1}H NMR (400 MHz, $CDCl_3$) *δ* 7.66 (s, 2H), 3.87 (s, 4H), 3.41 (s, 2H), 1.66 (s, 18H), 0.97 (s, 9H), 0.14 (s, 6H).); ^{13}C NMR (100 MHz, $CDCl_3$) *δ* 143.98, 120.35, 101.73, 88.78, 59.33, 48.10, 43.51, 30.17, 26.31, 16.68, 0.14, -4.29; MS (ESI+) m/z 445.0 $[M+2H]^+$.

N,N-bis((1-(tert-butyl)-1H-1,2,3-triazol-4-yl)methyl)prop-2-yn-1-amine (4): Compound (**3**) (720 mg, 1.62 mmol) was dissolved in tetrahydrofuran (13 mL), and tetrabutylammonium fluoride hydrate (850 mg, 3.25 mmol) was added, then the mixture was stirred at room temperature for 30 minutes. After the reaction solvent was removed under reduced pressure, Product was isolated using Silica-gel column chromatography (2% methanol / dichloromethane) to obtain 486 mg (91%) (4) as white solid. ^{1}H NMR (400 MHz, $CDCl_3$) δ 7.67 (s, 2H), 3.87 (s, 4H), 3.39 (d, J = 2.4 Hz, 2H), 2.28 (t, J = 2.4 Hz, 1H), 1.66 (s, 18H); ^{13}C NMR (100 MHz, $CDCl_3$) δ 143.62, 120.26, 78.83, 53.52, 59.19, 47.83, 42.21, 30.01; MS (ESI+) m/z 330.7 $[M+H]^+$, 352.7 $[M+Na]^+$.

General Synthesis of BTTA-PEGn-HTL: Compound (**4**) (300 mg, 0.91 mmol) and Azido-PEGn-HTL (1.09 mmol) were dissolved in acetonitrile (14 mL), $NaHCO_3$ (191 mg, 2.28 mmol), sodium ascorbate (45 mg, 0.2 mmol), $CuSO_4.5H_2O$ (57 mg, 0.2 mmol) was dissolved in water (7 mL) and added sequentially. The reaction mixture was stirred at room temperature for 1 hour. The organic layer obtained by extraction with ethyl acetate/water was passed through a Na_2SO_4 Pad to remove remaining moisture, and then the solvent was removed under reduced pressure. Product was isolated using Silica-gel column chromatography (2-4% methanol/dichloromethane) to obtain **BTTA-PEGn-HTL** as a yellow oil (Freezes at -20°C).

BTTA-PEG1-HTL: ^{1}H NMR (500 MHz, $CDCl_3$) δ 7.91 (s, 1H), 7.84 (s, 2H), 4.52 (s, 2H), 3.77 (d, J = 18.6 Hz, 7H), 3.48 (t, J = 6.6 Hz, 2H), 3.40 (t, J = 6.4 Hz, 2H), 1.71 (d, J = 7.2 Hz, 2H), 1.57 – 1.48 (m, 2H), 1.43 – 1.34 (m, 2H), 1.30 (dd, J = 14.8, 7.8 Hz, 2H).^{13}C NMR (125 MHz, $CDCl_3$) δ143.35, 142.41, 125.00 (s), 121.23 (s), 71.34 (s), 69.05 (s), 59.36 (s), 50.44 (s), 47.06 (s), 45.14 (s), 32.55 (s), 32.83, 30.16 (s), 29.42 (s), 26.70 (s), 25.45 (s) 22.65. MS (ESI+) m/z 535.3 $[M+H]^+$.

BTTA-PEG2-HTL: ^{1}H NMR (500 MHz, $CDCl_3$) δ 7.91 (s, 3H), 7.83 (s, 6H), 5.28 (s, 4H), 4.53 (t, J = 5.2 Hz, 7H), 3.87 (t, J = 5.2 Hz, 7H), 3.75 (s, 13H), 3.58 (dd, J = 5.4, 3.2 Hz, 7H), 3.55 – 3.47 (m, 14H), 3.41 (t, J = 6.6 Hz, 7H), 1.73 (dd, J = 14.4, 6.9 Hz, 7H), 1.59 – 1.51 (m, 8H), 1.42 (dt, J = 14.6, 7.2 Hz, 8H), 1.33 (dd, J = 15.0, 8.0 Hz, 8H). ^{13}C NMR (125 MHz, $CDCl_3$) δ 143.88 (s), 143.24 (s), 124.99 (s), 121.15 (s), 71.37 (s), 70.79 (s), 70.10 (s), 69.63 (s), 59.31 (s), 50.30 (s), 47.44 (s), 47.08 (s), 45.17 (s), 32.62 (s), 31.84 (s), 30.14 (s), 29.53 (s), 26.76 (s), 25.49 (s)22.68 (s). MS (ESI+) m/z 579.36 $[M+H]^+$.

BTTA-PEG3-HTL: ^{1}H-NMR (400MHz, $CDCl_3$) δ 7.93 (s, 1H), 7.85 (s, 2H), 4.54 (t, J = 5.2 Hz, 2H), 3.89 (t, J = 5.2 Hz, 2H), 3.79 (s, 2H), 3.76 (s, 4H), 3.64-3.50 (m, 10H), 3.44 (t, J = 6.8 Hz, 2H), 1.80-1.50 (m, 4H), 1.68 (s, 18H), 1.49-1.30 (m, 4H); ^{13}C-NMR (100 MHz, $CDCl_3$) δ 143.99, 143.33, 125.04, 121.19, 71.37, 70.82, 70.79, 70.66, 70.22, 69.68, 59.34, 50.334, 47.48, 47.12, 45.19, 32.67, 31.72, 30.18, 29.58, 26.82, 25.55, 22.79; MS (ESI+) m/z 624.0 $[M+H]^+$.

2-(4-((bis((1-(tert-butyl)-1H-1,2,3-triazol-4-yl)methyl)amino)methyl)-1H-1,2,3-triazol-1-yl)acetic acid (5): Compound (**4**) (300 mg, 0.91 mmol) and Azido-acitic-acid (137 mg, 1.36 mmol) were dissolved in acetonitrile (14 mL), $NaHCO_3$ (191 mg, 2.28 mmol), sodium ascorbate (45 mg, 0.2 mmol), $CuSO_4.5H_2O$ (57 mg, 0.2 mmol) was dissolved in water (7 mL) and added sequentially. The reaction mixture was stirred at room temperature for 1 hour and after complition of reaction, solvent was removed under reduced pressure and product was isolated using Silica-gel column chromatography (20-40% Methanol/Ethyl-acetate 1% acetic acid) to obtain BTTAA (**5**) as white solid. Obtained data was matched with the literature data[3]. ^{1}H NMR (500 MHz, D_2O) δ 7.97 (s, 2H), 7.89 (s, 1H), 5.02 (s, 2H), 3.83 (m, 6H), 1.62 (s, 18H).

2-(4-((bis((1-(tert-butyl)-1H-1,2,3-triazol-4-yl)methyl)amino)methyl)-1H-1,2,3-triazol-1-yl)-N-(4-hydroxyphenethyl)acetamide (BTTAT): In a 10 ml round bottom flask compound (5) (200 mg, 0.465

mmol), HATU (176 mg, 0.465 mmol) were taken with 2 ml of anhydrous DMF then 200 µL of DIPEA was added to the flask and stirred for 15 min at room temperature, after the colour turned deep brown, Tyramine (65 mg, 0.574 mmol) was added to the reaction mixtureand stirred at room temprature for another 2 hours, after the complition of reaction the organic layer obtained by extraction with ethyl acetate/water was passed through a Na_2SO_4 Pad to remove remaining moisture, and then the solvent was removed under reduced pressure. Product was isolated using Silica-gel column chromatography (4-6% methanol / Chloroform) to obtain 190 mg (75%) BTTAT as white powder. BTTAT ^{1}H NMR (500 MHz, $CDCl_3$) δ 7.80 (s, 2H), 7.53 (s, 1H), 6.85 (d, J = 8.0 Hz, 2H), 6.79 (d, J = 8.0 Hz, 2H), 6.06 (s, 1H), 4.94 (s, 2H), 3.76 (s, 2H), 3.73 (s, 4H), 3.47 (d, J = 5.8 Hz, 2H), 2.69 (t, J = 6.0 Hz, 2H), 1.67 (s, 18H). ^{13}C NMR (125 MHz, $CDCl_3$) δ 165.34 (s), 156.12 (s), 144.95 (s), 143.20 (s), 129.73 (s), 128.80 (s), 125.18 (s), 121.25 (s), 116.22 (s), 59.70 (s), 53.07 (s), 47.92 (s), 47.16 (s), 40.64 (s), 33.95 (s), 30.12 (s). MS (ESI+) m/z 550.33 $[M+H]^+$.

6-(5-methyl-2-oxoimidazolidin-4-yl)-N-(prop-2-yn-1-yl)hexanamide(Desthiobiotin-Alkyne): In a 10 ml round bottom flask *d*-desthiobiotin (100 mg, 0.46 mmol), HATU (143 mg, 0.512 mmol) were taken with 1 ml of anhydrous DMF then 120 µL of DIPEA was added to the flask and stirred for 15 min at room temperature, after the colour turned brown Propargyl-amine(30.84 mg, 0.560 mmol) was added to the reaction mixtureand stirred at room temprature for another 2 hours, after the complition of reaction the organic layer obtained by extraction with ethyl acetate/water was passed through a Na_2SO_4 Pad to remove remaining moisture, and then the solvent was removed under reduced pressure. Product was isolated using Silica-gel column chromatography (4-6% methanol / Chloroform) to obtain 105 mg (85%) Desthiobiotin-Alkyne as white powder. ^{1}H NMR (500 MHz, 5% CD_3OD in $CDCl_3$) δ 6.45 (s, 1H), 4.00 (dd, J = 4.9, 2.3 Hz, 2H), 3.86 – 3.77 (m, 1H), 3.64-3.69 (m, 1H), 2.22 (t, J = 2.4 Hz, 1H), 2.17 (t, J = 7.4 Hz, 2H), 1.68 – 1.58 (m, 2H), 1.65-1.3 (m, 8H), 1.09 (d, J = 6.5 Hz, 3H). ^{13}C NMR (125 MHz, 5% CD_3OD in $CDCl_3$) δ 173.63 (s), 164.24 (s), 71.13 (s), 55.95 (s), 51,35(s), 35.64 (s), 29.41 (s), 28.90 (s), 28.70 (s), 25.80 (s), 25.19 (s), 15.42 (s). MS (ESI+) m/z 252.17 $[M+H]^+$.

References in Supporting Information:

(1) Cai, B.; Kim, D.; Akhand, S.; Sun, Y.; Cassell, R. J.; Alpsoy, A.; Dykhuizen, E. C.; Van Rijn, R. M.; Wendt, M. K.; Krusemark, C. J. Selection of DNA-Encoded Libraries to Protein Targets within and on Living Cells. *Journal of the American Chemical Society* **2019**, *141* (43), 17057-17061

(2) Mishra, P. K.; Kang, M.-G.; Lee, H.; Kim, S.; Choi, S.; Sharma, N.; Park, C.-M.; Ko, J.; Lee, C.; Seo, J. K.; et al. A chemical tool for blue light-inducible proximity photo-crosslinking in live cells. *Chemical Science* **2022**, *13* (4), 955-966

(3) Besanceney-Webler, C.; Jiang, H.; Zheng, T.; Feng, L.; Soriano del Amo, D.; Wang, W.; Klivansky, L. M.; Marlow, F. L.; Liu, Y.; Wu, P. Increasing the efficacy of bioorthogonal click reactions for bioconjugation: a comparative study. *Angew Chem Int Ed Engl* **2011**, *50* (35), 8051-8056
