# Supplementary material for: GEN-Click: Genetically Encodable Click Reactions for Spatially Restricted Metabolite Labeling
Source: ACS Cent Sci. 2023 Jul 25;9(8):1650–7. doi: 10.1021/acscentsci.3c00511 (PMC10450880; doi:10.1021/acscentsci.3c00511)
Supplement: Supplementary file 2 — oc3c00511_si_002.pdf [file oc3c00511_si_002.pdf]

oc-2023-00511c.R1

Name: Peer Review Information for "GEN-Click: Genetically Encodable Click Reactions for Spatially Restricted Metabolite Labeling"

First Round of Reviewer Comments

Reviewer: 1
Comments to the Author
The authors describe a chemical biology technique for labeling cell surface proteins.

They first demonstrate with thorough experimentation that proteins bearing a ligand for copper (attached via surface tyrosines) is able to catalyze copper click reactions. In vitro and in lysate.

They extend this by using a membrane localized APEX construct to label the surface of cells with a ligand for copper. They show that proximity labeling is required to maintain enough cell surface copper to perform click reactions onto metabolically incorporated azido sialic acids.

They then show that the proximity labeling can be directed by a nanobody to a HA expressing transmembrane domain.

The authors also showed that alkynyl choline can be used as a metabolic handle, which can be clicked onto azido coumarin following deposition of soluble copper via proximity labeling.

Finally, the authors used a two-cell co-culture to visualize labeling in trans. This experiment showed biotinylation more broadly than might have been expected. Here the cell membranes of the APEX expressing cells were broadly labeled, rather than just the cell-cell junctions. From this, the authors hypothesize that this results from exchange of metabolites from cell to cell which leads to azido sialoglycoproteins on the surface of the APEX expressing cell and subsequent mis-labeling.

Manuscript strengths: Overall, there are some strong themes that have been explored in this manuscript. The use of proximity labeling to perform localized chemical reactions has never been demonstrated and this concept could prove to have many applications. The combination of metabolic incorporation with localized labeling is also interesting.

The work demonstrated is generally well done, with suitable controls.

Weaknesses: The data shown in the SI is not particularly strong. The AHA expt and the “receptor” experiment in particular are not convincing – why is biotinylation so low?

One of the key strengths in this work is the use of proximity labeling, however, because both the metabolic incorporation and the APEX-TM are broadly incorporated across the whole

membrane the same result could be achieved through a simple SPAAC reaction (i fact, I think all images shown could be obtained via this method). It is puzzling why APEX/HRP was not localized to a more specific receptor to see a subset of the cell membrane labeled. It would be powerful to show metabolic incorporation everywhere, but only those glycoproteins be labeling adjacent to a particular receptor. The authors also don't target a specific biological problem. The method is clearly useful but hasn't really been deployed to a great extent in this study.

In the final experiment, as far I can see, there is no image showing only the cell-cell junction labeled (as would be expected). I think more data is needed here to say anything convincing about metabolite transfer. Is there a time dependence on this transfer? Are other metabolites transferred (alkynyl choline also)? Are there metabolites free in the medium (direct transfer as pictured or in the media through passive/active transport)?

It is also surprising not to see more labeling on the azide bearing cell. APEX has been shown to broadly label cells in trans by Geri et al. (Science, 2020), but in this case, no labeling is really seen at all on the azide bearing cell.

This final expt is really the meat of the study so a little more work is required to flesh out what exactly is going on and perhaps make some statement about the implications of what has been found.

The microscopy brightfield images are not great, some are actually very hard to read at all.

Finally, in this reviewer's opinion, the manuscript would benefit from some editing. There are discussions in the paper that only confuse the actual data being presented. I don't believe there is a need to discuss the halotag expt, the AHA expt, the receptor experiment (as it didn't work very well), or the alternative use of HRP instead of APEX. These add a lot of acronyms and made it harder for me to understand the core of the study.

Minor errors.
As far as I am aware ManAz is metabolized into azido sialic acid before glycoprotein incorporation. This is missing from the figures, which seem to suggest that the ManAz is incorporated directly.

There are far too many acronyms in this study. It was quite difficult to read as I was constantly checking what each meant. I would suggest that at the very least, DTB just be written out as desthiobiotin.

Figure s4: I assume that TM-HRP has a myc tag? That isn't clear from the caption or figure.

Page 2 line 39 "conjugated proteins a streptavidin-HRP" should this be via

Figure 3C: Ascorbate should read NaAsc or Sodium Ascorbate or Na Ascorbate (change throughout).

Figure 3D. DPB lane is shown twice. H2O2 should be superscripted (fix super/subscripts throughout)

Figure s6: are the images mixed up here? Images show sample 1 without and GFP bu the legend suggests Sample 1 should have all components. Authors need to check this. Further, there are many small errors in formatting and spacing in the figure.

Figure s6: the biotinylation is very weak in the positive lane. Can this be explained?

In the procedures: "Milipore" should be Millipore

Reviewer: 2
Comments to the Author
This manuscript by Mishra et al. describes a novel strategy to perform the CuAAC reaction in living cells with a genetically encoded tag. Such a spatially restricted click reaction benefits the profiling of newly synthesized proteome and post-translational modifications (PTMs) in a subcellular specific manner. Overall, the idea is inspiring and the authors have provided experimental data demonstrating the feasibility of this novel approach. The following issues need to be addressed before the manuscript could be considered for publication at ACS Central Science.

1. The authors first introduced a HaloTag-based strategy, but quickly dismissed the idea because the catalytic efficiency appeared low, which the authors attributed to the "the proteinaceous environment of HaloTag" that "inhibits BTTA catalytic activity or enforces a mononuclear state of Cu-BTTA". However, such statement is not supported by experimental evidence. Further, the remaining parts of the manuscript focus exclusively on the peroxidase-based strategy, which is conceptually quite different from the HaloTag approach. Such a transition in the main text could be confusing to the readers. It is this referee's opinion that the HaloTag part should be removed from the main text.
2. The authors report the observation of catalytic activity toward click reaction in "non-modified BSA" lacking the BTTA ligand (Figure 2B, brown trace). The authors should elaborate on the reason behind this background catalysis. Do these data suggest that free copper ions are capable of catalyzing the click reaction? There is a clear dose-dependence in the catalytic efficiency. What is the catalytic activity in the absence of BSA? More experimental data with additional controls are needed to clarify this issue.
3. Page 2, line 49: with the similarity in chemical structures of BTTAT and the commercially available ligand BTTAA, why is BTTAT compared with THPTA instead of BTTAA?

4. To better demonstrate this GEN-Click system as an efficient platform for examining the metabolite-transferring events, the authors are suggested to provide at least one more metabolite-transferring case detected by the GEN-Click system.

Author's Response to Peer Review Comments:

Summary of our revision

Major Concerns	Related Reviewers comment	Our Response (**Additional Results**)
Low biotinylation	Reviewer #1-1<br>Reviewer #1-4	Owing to the requirement physical interaction of "Cu-BTTAT-Tyr" with ManAz and alkyne, Gen-click has low biotinylation but can label physically interacting proteins with accuracy, whereas APEX can generate biotin-phenoxyl radicals and carry out spray type biotinylation, which ensures vigorous and diffusive labeling.<br><br>**GEN-Click vs APEX2 biotin-labeling in ligand-receptor labeling result** (Figures R1 and 2)
More experiments needed for metabolite transfer and contact site related work.	Reviewer #1-3<br>Reviewer #2-4	We have included additional experiments related to metabolite transfer and contact site labeling:<br>1) **Alkyne-Choline metabolite transfer experiment** (Figures R3, R15).<br>2) **Validation of secreted ManAz in the media from ManAz-treated cells** (Figure R4).<br>3) **Time dependent metabolite transfer** in co-culture cells (Figure R5).<br>4) **Metabolite transfer under the gap junction inhibitor (Carbenoxolone) treatment** (Figure R7).
Use of excessive acronyms which made understanding the core of study difficult.	Reviewer #1-7	We have minimized use of various acronyms (i.e. DTB: desthiobiotin, HA-FB: HA-Frankenbody etc.) ensure a straightforward understanding of the study.

Reviewer(s)' Comments to Author:

Reviewer #1

Recommendation: Reconsider after major revisions noted.

Comments:

The authors describe a chemical biology technique for labeling cell surface proteins. They first demonstrate with thorough experimentation that proteins bearing a ligand for copper (attached via surface tyrosines) is able to catalyze copper click reactions. In vitro and in lysate. They extend this by using a membrane localized APEX construct to label the surface of cells with a ligand for copper. They show that proximity labeling is required to maintain enough cell surface copper to perform click reactions onto metabolically incorporated azido sialic acids. They then show that the proximity labeling can be directed by a nanobody to a HA expressing transmembrane domain.

The authors also showed that alkynyl choline can be used as a metabolic handle, which can be clicked onto azido coumarin following deposition of soluble copper via proximity labeling.

Finally, the authors used a two-cell co-culture to visualize labeling in trans. This experiment showed biotinylation more broadly than might have been expected. Here the cell membranes of the APEX expressing cells were broadly labeled, rather than just the cell-cell junctions. From this, the authors hypothesize that this results from exchange of metabolites from cell to cell which leads to azido sialoglycoproteins on the surface of the APEX expressing cell and subsequent mis-labeling.

Manuscript strengths: Overall, there are some strong themes that have been explored in this manuscript. The use of proximity labeling to perform localized chemical reactions has never been demonstrated and this concept could prove to have many applications. The combination of metabolic incorporation with localized labeling is also interesting.

The work demonstrated is generally well done, with suitable controls.

(Reviewer #1-1) Weaknesses: The data shown in the SI is not particularly strong. The AHA expt and the “receptor” experiment in particular are not convincing – why is biotinylation so low?

(Response) First, we would like to thank the reviewer for their valuable time in reviewing this manuscript. We acknowledge and agree with the observation that the samples with AHA showed lower levels of labeling in comparison to the ManAz sample. Therefore, we would like to propose two potential explanations for this disparity:

1. Accessibility of AHA: Owing to the glycans' extensive shielding, the incorporated AHA in cell surface proteins may have limited accessibility (**Figure R1A**). This reduced accessibility could hinder the reaction with Cu-BTTAT-modified proteins, resulting in lower levels of biotinylation. In contrast, azido-sialic acid, present at the tip of the glycan structure, is more exposed to the solvent and can readily react with Cu-BTTAT-modified proteins, thus potentially explaining the stronger biotinylation observed in the ManAz sample (**Figure R1B**).

2. <u>Toxicity</u>: AHA has been reported to demonstrate significant toxicity in mammalian cells [PMID: 27572480] and can affect the expression of APEX2, the enzyme responsible for biotinylation. In our AHA-incubated samples, we observed some impact on APEX2-TM expression, which could have contributed to the lower biotinylation levels (**Figure R1D**). However, ManAz incorporation did not show any significant difference in APEX2-TM expression, potentially leading to stronger biotinylation (**Figure R1C**).

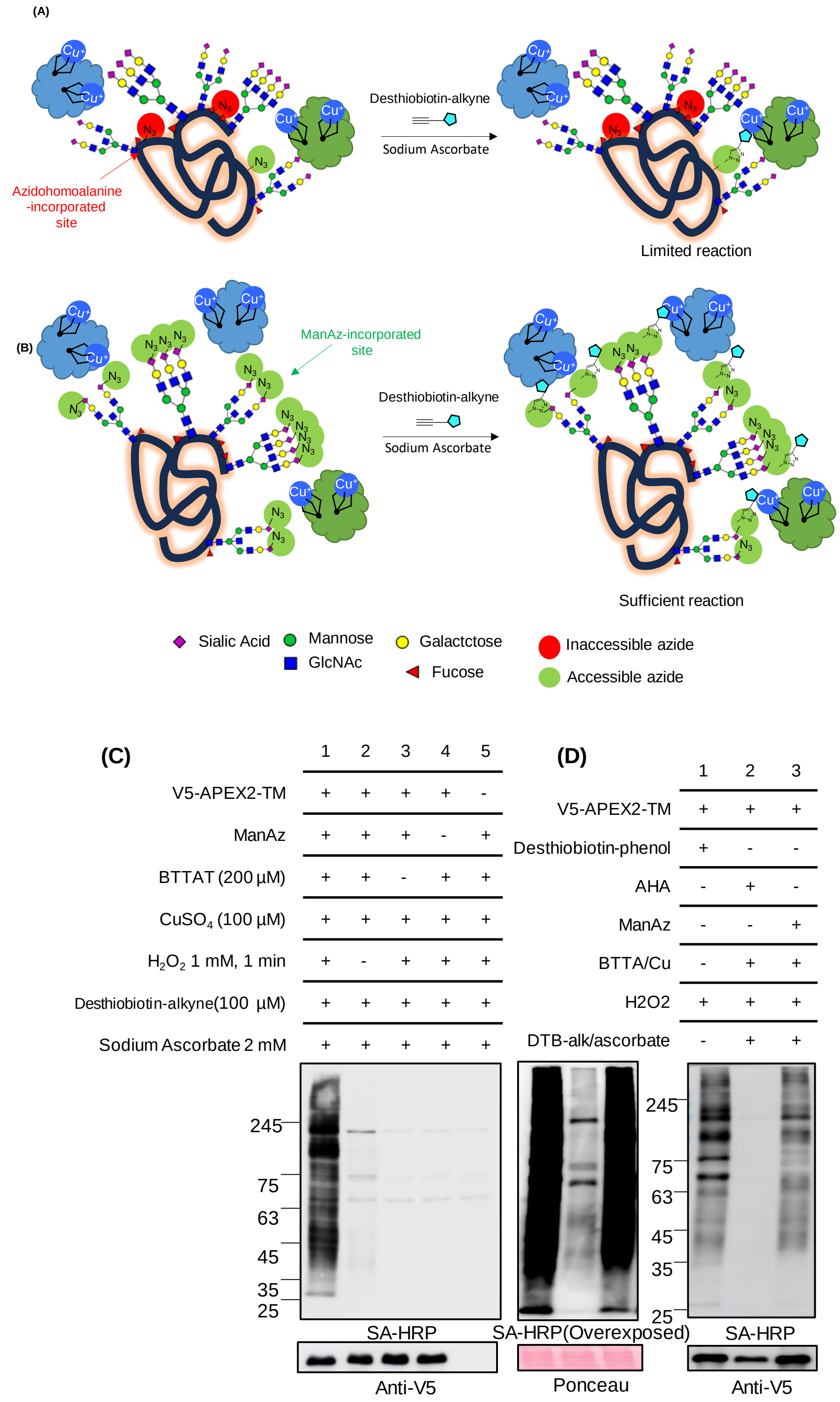

Figure R1. Reason for the low biotinylation activity of GEN-Click with Azidohomoalanine (AHA).

In our ligand-receptor (Frankenbody-APEX2::HA-mCherry-TM) experiment, we implemented a thorough washing procedure to remove any unbound Frankenbody-APEX2 from the extracellular matrix. Our aim was to ensure that the ligand-APEX protein (Frankenbody-APEX2) primarily localizes at the receptor protein (HA-mCherry-TM). Using this protocol, we could achieve specific biotinylation of the receptor protein and its neighboring interacting partners as shown in **Figure R2**.

The lower biotinylation observed with GEN-Click in the ligand-receptor experiment may also be attributed to the relatively low copper concentration remaining at the ligand protein after the thorough washing step. This limitation could potentially be addressed in future experiments by using higher copper binding catalysts as commented in the Discussion part of our manuscript. We appreciate your careful consideration of the experimental design and data interpretation.

(Reviewer #1-2) One of the key strengths in this work is the use of proximity labeling, however, because both the metabolic incorporation and the APEX-TM are broadly incorporated across the whole membrane the same result could be achieved through a simple SPAAC reaction (in fact, I think all images shown could be obtained via this method). It is puzzling why APEX/HRP was not localized to a more specific receptor to see a subset of the cell membrane labeled. It would be powerful to show metabolic incorporation everywhere, but only those glycoproteins be labeling adjacent to a particular receptor. The authors also don't target a specific biological problem. The method is clearly useful but hasn't really been deployed to a great extent in this study.

(Response) We appreciate this valuable comment. We understand your point that localizing APEX/HRP to a specific receptor would allow for the selective labeling of a subset of the cell membrane adjacent to that receptor, which might be valuable to study the specific glycoprotein-protein interactions at the cell surface.

In our current study, we focused on the construction of GEN-click system, demonstration of its reliability in live cell experiments, and determination of its novelty compared to the conventional methods (e.g., proximity labeling). During the revision period, we tested whether GEN-click labeling has a better labeling specificity than APEX labeling. For this experiment, we utilized our ligand-receptor ID system using APEX2-Frankenbody (ligand) and HA-mCherry-TM (receptor protein). In this reaction, APEX2-Frankenbody conducted two different biotin labeling reaction, respectively: desthiobiotin-alkyne labeling on the ManAz-incorporated protein after GEN-Click modification or conventional phenoxyl radical labeling by using desthiobiotin-phenol (DBP) [PMID: 28156110]. As shown in **Figure R2**, we found that very selective biotin labeling of receptor protein was observed in Frankenbody-APEX2:GEN-Click labeling while Frankenbody-APEX2:DBP labeling less specific biotin labeling of the receptor protein (**Figure R2C**), which makes our method superior to the conventional APEX method.

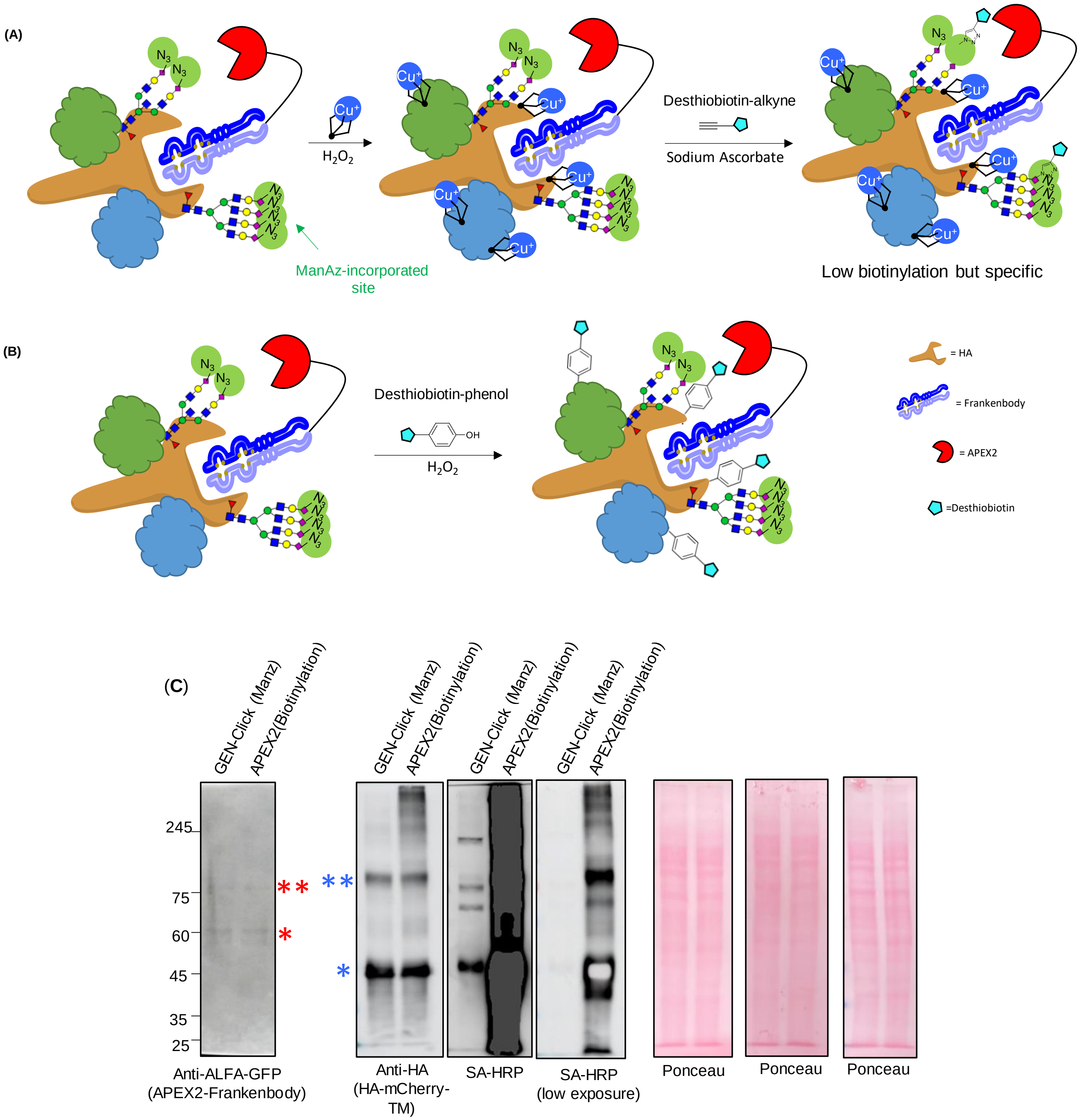

Figure R2 (related to Figure S5): GEN-Click vs APEX2 labeling. (**A**) Scheme of GEN-Click mediated biotinylation of receptor protein. (**B**) Scheme of APEX-mediated desthiobiotin-phenoxyl radical labeling of receptor protein and other proteins. (**C**) Western blot analysis results of HA-mCherry-TM expressing cells. ManAz (100 µM overnight incubation) was used as azide-incorporating block, APEX2-Alfa-Frankenbody was used as ligand on cell surface, and Desthiobiotin-alkyne was used to perform click reaction. For APEX2's phenoxyl radical labeling, 250 µM Desthiobiotin-Phenol (DBP) and 1 mM H_2O_2 were incubated for 1 min and washed thrice with 5 mM NaN_3, 10 mM sodium ascorbate, and 10 mM Trolox. After the labeling, cells were lysed with RIPA lysis buffer. In the western blot results, red asterisk marks the ligand protein and blue asterisk marks receptor proteins. Double asterisks mark crosslinked complex in presence of H_2O_2.

We believe that this result shows the significant potential of the GEN-Click method in identifying "specific" ligand-receptor interactions **(Figure R2A).** The identification of ligand-receptor interactions is crucial for understanding ligand signaling and the development of ligand-based therapeutics. As per the reviewer's valuable comment, we believe that Gen-Click can serve as a valuable tool for studying various interesting questions related to cis- or trans-glycoprotein-protein interactions, particularly at the neuronal cell surface. Proximity labeling has been widely used in these fields [PMID: 36742360, PMID: 27565350, PMID: 33067390], and we anticipate that our GEN-Click approach can contribute to further advancements in this area of research in the future.

Using GEN-Click, we also confirmed metabolite transfer event of Mannose-Azide (ManAz) or Azido-Choline at the cell-cell interface, rather than through the secretion pathway **(Figure R3, 4)**. We believe that these biological findings by GEN-Click will be meaningful for the biologists who study the metabolite transfer event through the gap junction. We further described the implications of our method and findings in the response to the comment 5 (**Reviewer #1-5**).

(**Reviewer #1-3**) In the final experiment, as far I can see, there is no image showing only the cell-cell junction labeled (as would be expected). I think more data is needed here to say anything <u>convincing about metabolite transfer</u>. Is there a time dependence on this transfer? Are other metabolites transferred (alkynyl choline also)? Are there metabolites free in the medium (direct transfer as pictured or in the media through passive/active transport)?

(Response) Thank you for this excellent suggestion! During the revision, we conducted the suggested experiments, including 1) the transfer of other metabolites such as Alkyne-Choline, as well as validation of metabolite transfers via 2) media- and 3) time-dependent transfer. Below, we provide additional details on these results**.**

<u>Alkynyl-Choline transfer</u>:

Following the valuable suggestion of the reviewer, we performed an experiment involving the transfer of Alkyne-Choline, and the results were consistent with the findings of ManAz transfer. In this experiment, we co-cultured APEX2-TM transfected cells with alkyne-choline (Propargyl-Choline) treated cells overnight (~12 h). After co-culturing, we treated azido-coumarin for the visualization of the transferred Prop-Chol population in APEX2-TM cells after BTTAT-Cu labeling under the non-permeabilization condition for the preservation of phospholipids in the sample (**Figure R3A**). Although the fluorescence intensity of azido-coumarin was lower than that of Streptavidin-AF647 used in the ManAz transfer experiment to visualize biotinylated azido sialic acid, the azido-coumarin signal was clearly shown in the APEX2-TM cells, indicating successful transfer of Alkyne-Choline between cell junctions (**Figure R3B**).

These results demonstrate that our GEN-Click method is useful for visualizing cell-to-cell transfer events of other metabolite surrogates, such as Alkyne-Choline. Furthermore, we believe that this additional result expands our understanding of metabolite transfer events in mammalian cells. Again, we greatly appreciate the valuable suggestion from the reviewer.

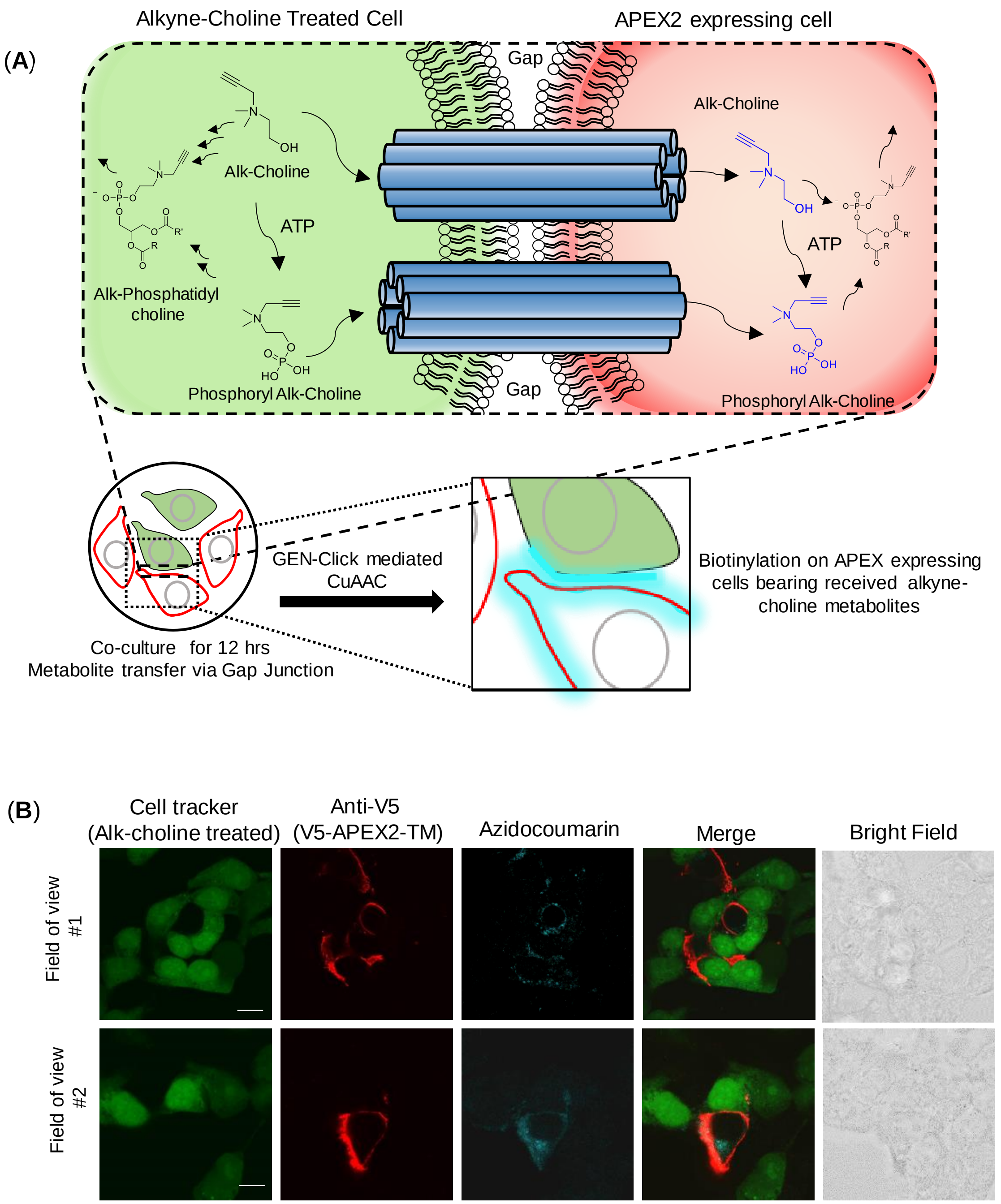

Figure R3 (related to Figure S7) (**A**) Scheme of fluorescence imaging of alkyne-choline transferring event at the cell-cell contact site using GEN-Click (**B**) Confocal images of Alkyne-Choline metabolites being transferred to adjacent contact cells in a co-cultured condition. Expression levels of APEX2-TM confirmed by anti-V5/mouse-AF568 antibody. BTTAT-Cu labeled Alkyne-Choline visualized with copper-clicked azidocoumarin (Ex=404/Em 477). Scale Bar 10 µm.

Media transfer:

As per the reviewer's suggestion, it is plausible that azido-mannose (ManAz) or its derivatives can be secreted into the media and taken up by APEX-TM cells, where it can be utilized as a precursor for glycosylation. To test this hypothesis, we collected the media that was secreted overnight from cells treated with ManAz, which had

been pre-incubated with 50uM ManAz for 12 h. Then, the collected media was treated to the APEX2-TM cells and further incubated for 12h. After the media incubation, APEX2-TM cells were labeled with BTTAT-Cu and biotin-alkyne was added for the possible in situ click reaction with BTTAT-Cu modified proteins at the cell surface (**Figure R4**). If the cellular uptake of secreted ManAz or its derivatives actively occurred in APEX2-TM cells, we would expect the generation of biotinylated proteins in this sample. However, as depicted in **Figure R4**, we found that only endogenous biotinylated proteins were detected in the Streptavidin-HRP (SA-HRP) western blot analysis of the media transfer sample (lane 2), which is similar to the result of the normal media treatment sample (lane 3). As a control, intensively biotinylated proteins were generated in the co-culture sample (i.e., ManAz-treated cell + APEX-TM cells, lane 1), indicating that mannose-azide transfer via media is likely negligible compared to direct cell-to-cell transfer.

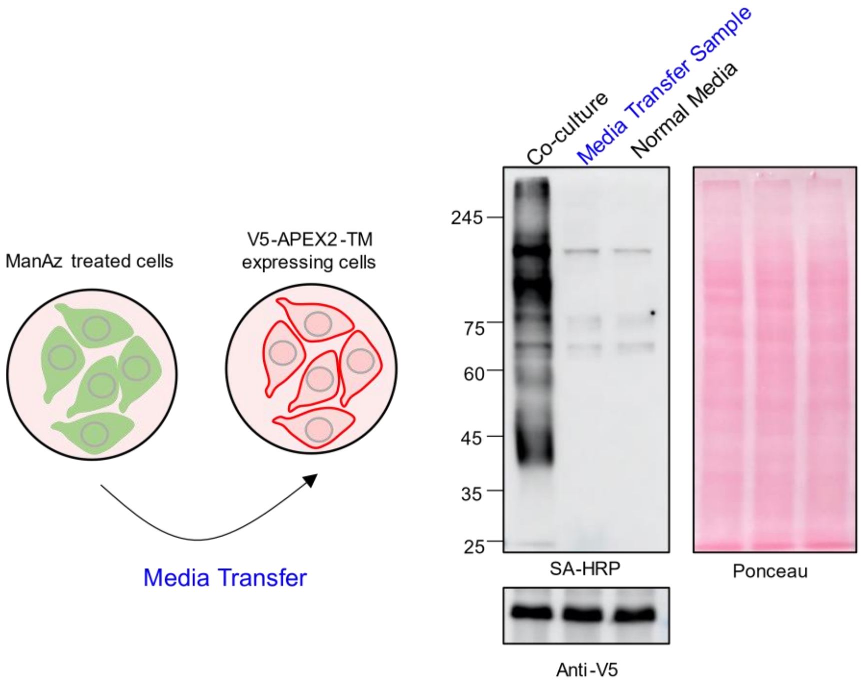

Figure R4. Validation of media transfer of mannose-azide (ManAz) (related to Figure S8A) (**Left**) Scheme of media transfer experiment (lane 2 of B). (**Right**) Western blot analysis of GEN-Click biotinylated proteins in three different samples. Lane 1: GEN-Click labeled sample from normal co-cultured APEX2-V5-TM expressing cells and ManAz-treated cells Lane 2: GEN-Click labeled sample of APEX2-V5-TM expressing cells which were treated with incubated media of ManAz-labeled cells for overnight. Lane 3: GEN-Click labeled sample of APEX2-V5-TM expressing cells grown in normal media (DMEM). In the result, Streptavidin-HRP showed desthiobiotin-alkyne labeled proteins and anti-V5 showed the expression level of V5-APEX2-TM.

Time-dependent metabolite transfer:

As per the reviewer's suggestion, we have performed experiments with incubation of APEX2-TM expressing cells with ManAz incorporated cells with incubation times of 4, 8, and 12 h. and we could observe that biotinylation due to metabolite exchange increased consistently with time (**Figure R5**).

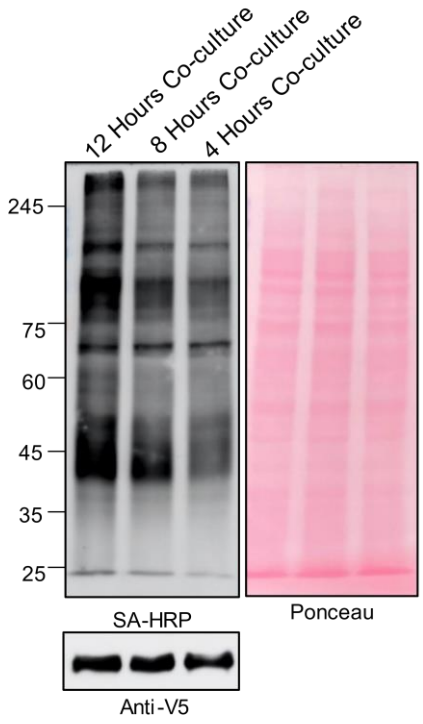

Figure R5 (Related to Figure S8B). Time dependent metabolite transfer. Western blot analysis of GEN-Click biotinylated proteins in three different co-culture samples. In these samples, co-culture was done in a time dependent manner for 12, 8 and 4 hours respectively and GEN-Click system was applied to visualize transferred metabolites via biotinylation on azido-sialic acid. In the result, Streptavidin-HRP showed desthiobiotin-alkyne labeled proteins and anti-V5 showed the expression level of V5-APEX2-TM.

(**Reviewer #1-4**) It is also surprising not to see more labeling on the azide bearing cell. APEX has been shown to broadly label cells in trans by Geri et al. (Science, 2020), but in this case, no labeling is really seen at all on the azide bearing cell.

(**Response**) Thank you for your valuable comment. We sincerely apologize for any confusion caused by the unclear presentation of our figure. In our revised Figure 4 (now **Figure R6**), we have included two types of GEN-Click labeling: contact-site labeling (Type 1 labeling pattern) and metabolite transfer labeling (Type 2 labeling pattern). In the case of Type 1 labeling, we clearly demonstrated that the surface of the azide-bearing cell, which represents the cell-cell contact site, was biotinylated by GEN-Click (see **Figure R6A**). Additionally, we want to emphasize that the azide-bearing cell surface can also be labeled by GEN-Click during Type 2 labeling, as indicated in the revised figure in our manuscript (**Figure R6B**).

In our ligand-receptor experiment, we observed significant labeling of proteins on the ManAz-bearing cells by ligand-APEX2 protein using Gen-click **(Figure R2).** Thus, we believe that there are no physical limitations to trans-labeling with GEN-Click at the cell-cell interface. We sincerely appreciate the reviewer for bringing this error to our attention.

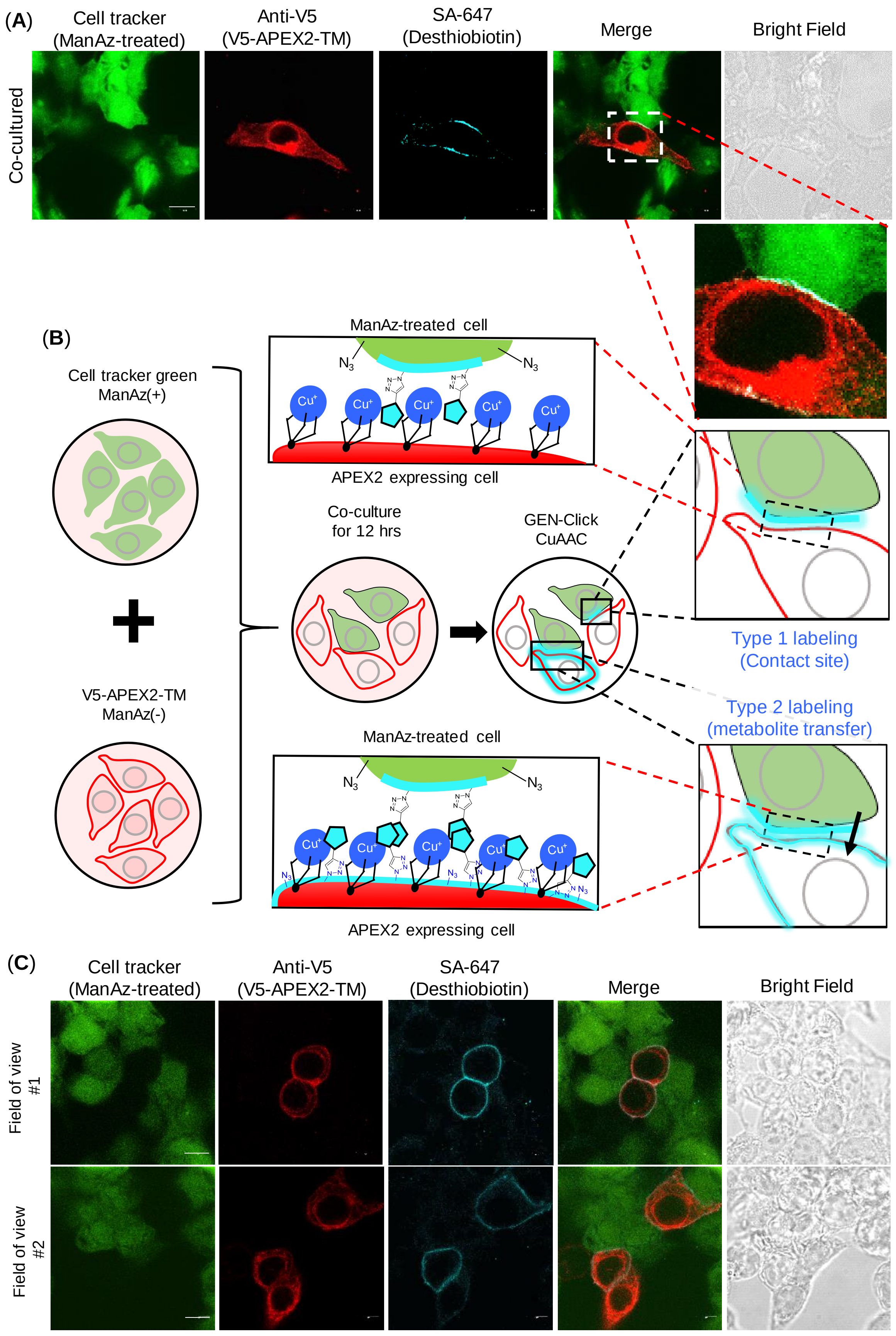

Figure R6 (related to Figure 4) Corrected reaction scheme of GEN-Click labeling at the cell-cell interface of co-cultured cells.

(**Reviewer #1-5**) This final expt is really the meat of the study so a little more work is required to flesh out what exactly is going on and perhaps make some statement about the implications of what has been found.

(**Response**) We appreciate the reviewer's comment. Since metabolite transfer events of mannose -azide (Figure **R5, 6, 7**) and alkyne-choline (**Figure R3**) via direct cell-cell interfaces using the GEN-Click system, we anticipate that our method can be further utilized to investigate various metabolite transfer events involving azido- or alkyne-moiety containing metabolite precursors [PMID: 30715839] in the future. Currently, the measurement of these metabolite transfer events via gap junctions is indirect and relies on the use of various fluorescent dye molecules (e.g., lucifer yellow, 6-carboxyfluorescein [PMID: 22796188]). Therefore, our system holds the potential to directly visualize metabolite transfer events and their functional utilization (i.e., glycosylation) in recipient cells. From a method development standpoint, we believe that GEN-Click can be a valuable approach for visualizing transferred metabolites at the cell-cell contact interface. From a biological perspective, only a limited number of metabolites (e.g., ATP, NAD+, Glutamate, Glutathione, PGE2 [PMID: 22796188]) have been characterized as being transferred at the cell-cell interface via gap junctions. Our study suggests that mannose and choline (or their derivatives) can be added to this list. Recently, metabolite transfer events through gap junction proteins have been recognized as a tumor survival pathway under mutagenesis conditions [PMID: 36107487]. We anticipate that our system can be employed for inhibitor screening of this metabolite transfer event.

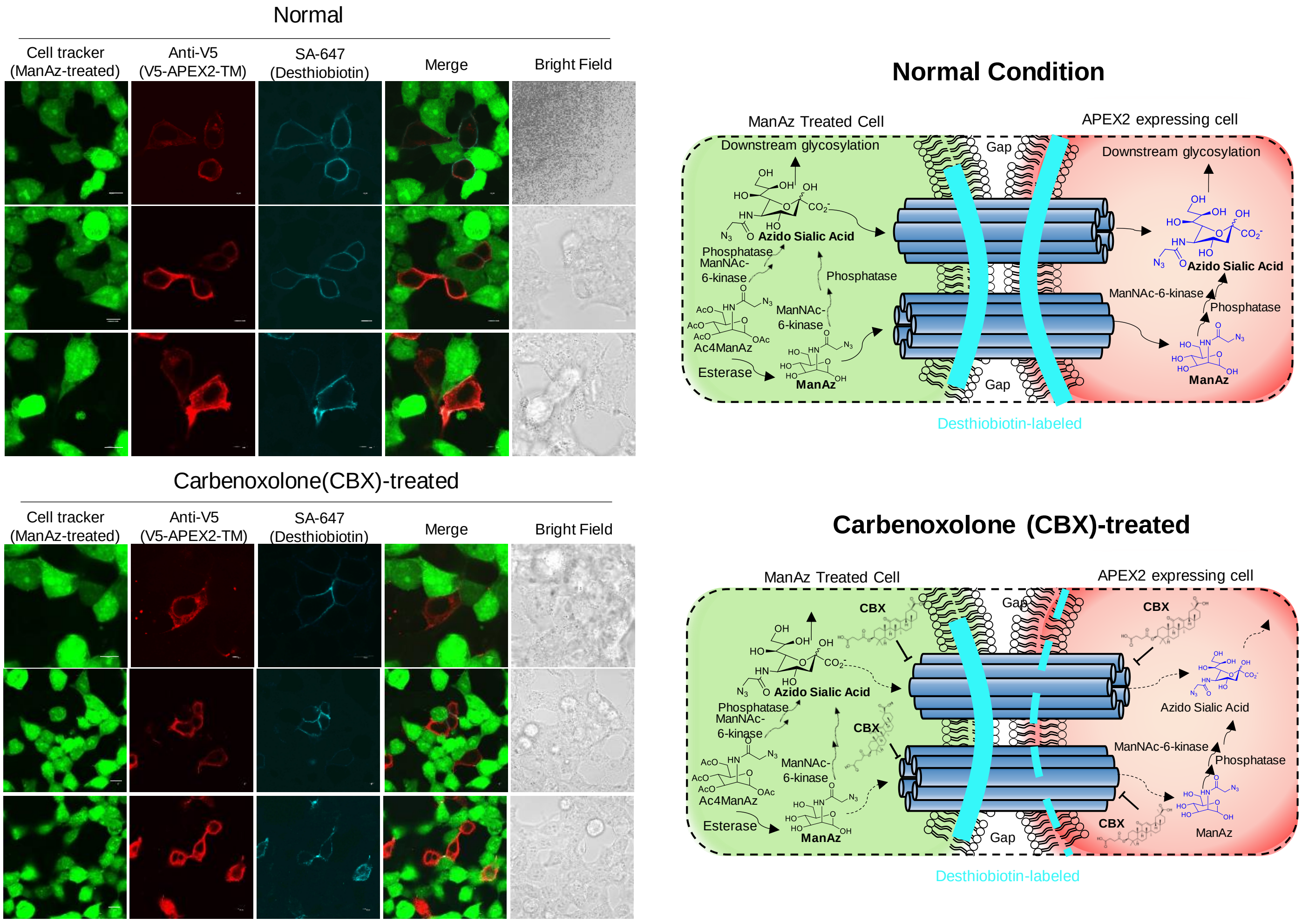

Figure R7 (related to Figure S8C) (Left) Gap junction inhibitor treated result. For CBX-treated samples, CBX (100 μM) were treated for 12 hrs on the co-cultured of ManAz-treated cells (marked with Cell Tracker, green fluorescence) and V5-APEX2-TM expressing cells (marked with anti-V5/Mouse-568 antibody). Desthiobiotin-

labeled molecules were visualized with Streptavidin-AF647 (SA-647). Control cells were cultured with normal media for 12 h. Both cells were treated with BTTAT/Cu and desthiobiotin-alkyne as described in the Material and Methods section of the manuscript. Scale bar 10 µm (**Right**) Schematic representation of inhibition of ManAz transfer to adjacent cells by CBX. Transferred metabolites were marked with blue color.

For instance, we have tested Carbenoxolone (CBX), a gap junction protein inhibitor, in our co-culture system (ManAz-treated cells + APEX-TM expressing cells). As shown in **Figure R7**, treatment with 100 µM Carbenoxolone, affected the evenly distributed GEN-Click labeling pattern (Type 2 labeling) on the APEX2-TM cells. This result supports that GEN-Click can be a useful tool for inhibitor screening of metabolite transfer in cancer cells.

From a chemical perspective, we also believe that our work significantly broadens the substrate scope of proximity labeling. In comparison to the currently limited range of substrates for proximity labeling (such as proteins, RNA, and DNA) [PMID: 34663510], the utilization of GEN-Click enables an expanded substrate scope. It allows for the labeling of azido-incorporated proteins (e.g., AHA) as well as azido-incorporated metabolites (such as glycans and phospholipids), utilizing various azido- or alkyne-bearing small biomolecules that can be taken up via salvage path-ways in live cells.

We have discussed these potential implications of our method in the revised manuscript's Discussion section. We thank the reviewer for providing this constructive comment!

(**Reviewer #1-6**) The microscopy brightfield images are not great, some are actually very hard to read at all.

(**Response**) Thank you for the valuable comment. Brightfield Images have been adjusted for brightness and contrast for better visualization (**Figure R8**).

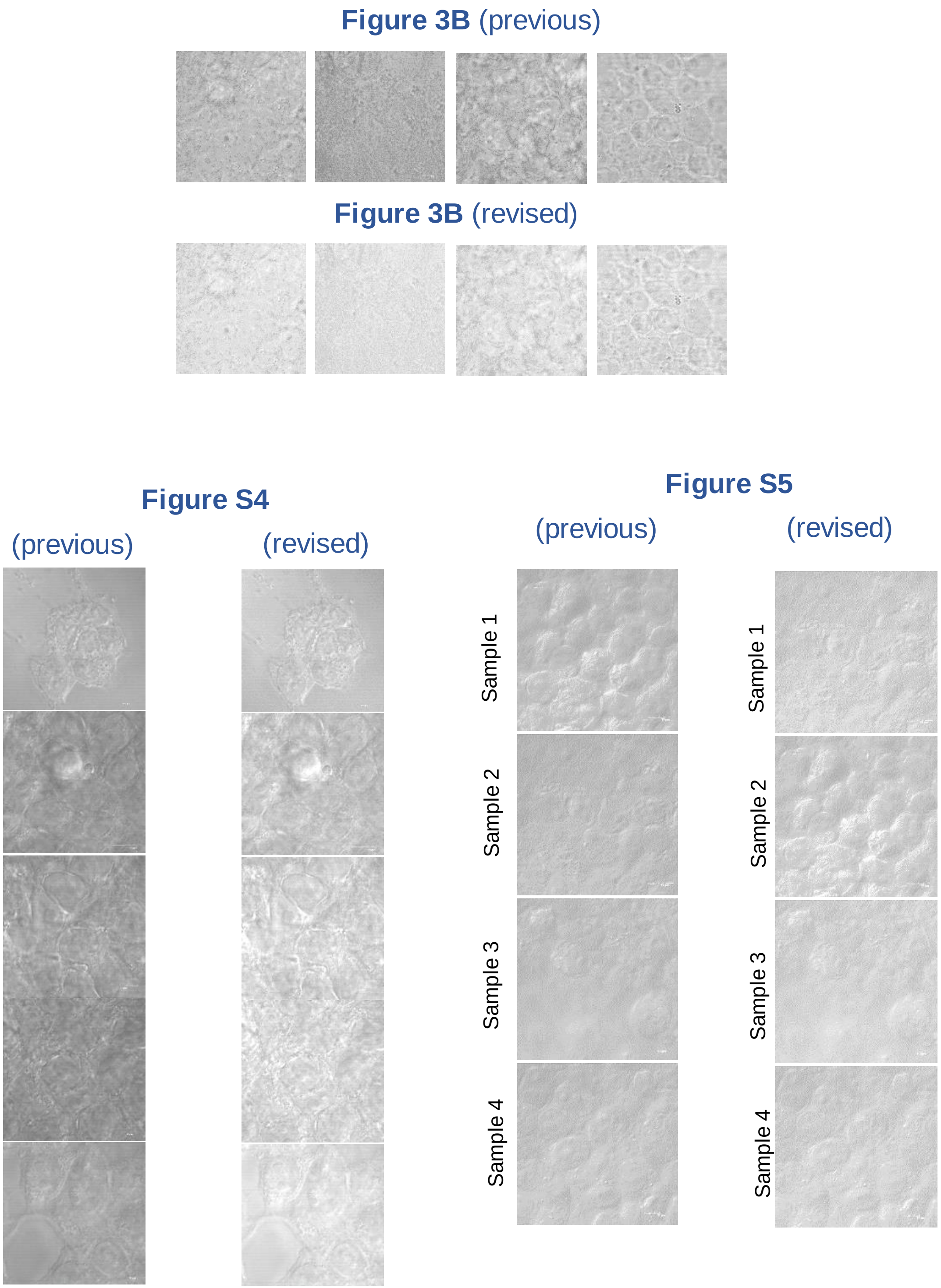

Figure R8. Bright Field image correction.

(**Reviewer #1-7**) Finally, in this reviewer's opinion, the manuscript would benefit from some editing. There are discussions in the paper that only confuse the actual data being presented. I don't believe there is a need to discuss the halotag expt, the AHA expt, the receptor experiment (as it didn't work very well), or the alternative use of HRP instead of APEX. These add a lot of acronyms and made it harder for me to understand the core of the study.

(**Response**) We would like to express our gratitude to the reviewer for this valuable suggestion. We agree that many of our results seem overlapped with each other (e.g., APEX/HRP, AHA/ManAz). As your suggested, we deleted previous Fig. S5 result (APEX2/AHA result) which was overlapped with Fig. S4 result. We also shorten the description of Fig. S3 (BSA-GEN-Click cell lysate experiment) in the main text. However, we believe that other results which were conducted under different conditions can enhance the robustness and reliability of our novel method. While we would like to retain this data in our manuscript as it does not contradict other findings, we are open to following the reviewer's or editor's guidance once a decision is made.

Additionally, we agree with the reviewer that the HaloTag experiment is not essential for our current study since it was not selected as part of the GEN-Click method in our work. However, we believe that this part will be particularly interesting for protein engineering society. Based on our crystal data (PDB ID: 8J1O), we observed significant intermolecular interactions between the triazole ring of BTTA and cell surface residues of HaloTag (e.g., hydrogen bonding, pi-pi interaction; **Figure R9**). Further investigations could be intriguing for the development of a new genetically engineered click reaction approach utilizing a HaloTag mutant that preserves the catalytic activity of Cu-BTTA. Currently, many chemists are interested in the development of design or engineering biocatalyst, we believe that the HaloTag aspect may still hold relevance for this community. While we have included a discussion on the future direction of HaloTag engineering in the revised manuscript, we will follow the decision of the editor and reviewer if the removal of this part is suggested. We have also replaced many acronyms with their full forms to facilitate a better understanding of our study. We sincerely appreciate this helpful comment.

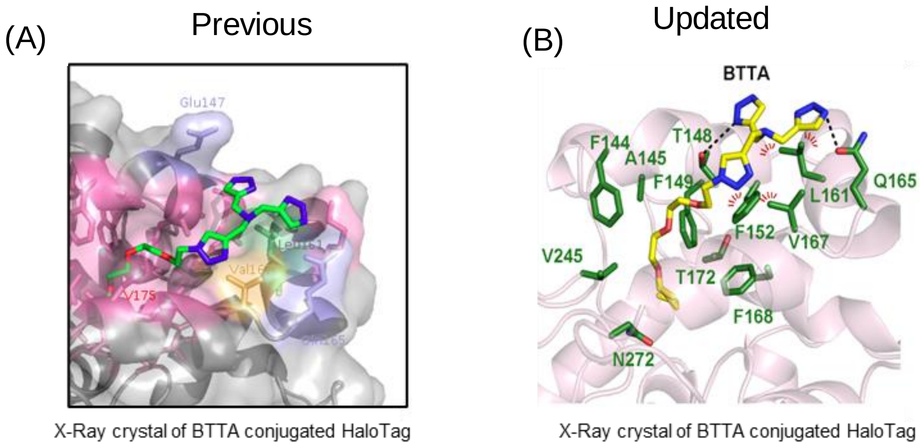

Figure R9. Crystal structure of HaloTag-BTTA (PDB ID: 8J1O): (A) previous version (B) revised version. The newly added picture shows pi-pi and hydrogen bond interactions of BTTA moiety with surface residues.

Minor errors

(Reviewer #1-8) As far as I am aware ManAz is metabolized into azido sialic acid before glycoprotein incorporation. This is missing from the figures, which seem to suggest that the ManAz is incorporated directly.

(**Response**) We have added the chemical structure of azido-sialic acid as a representative metabolite that can be incorporated on the glycoproteins in figures, also commented in the main text.

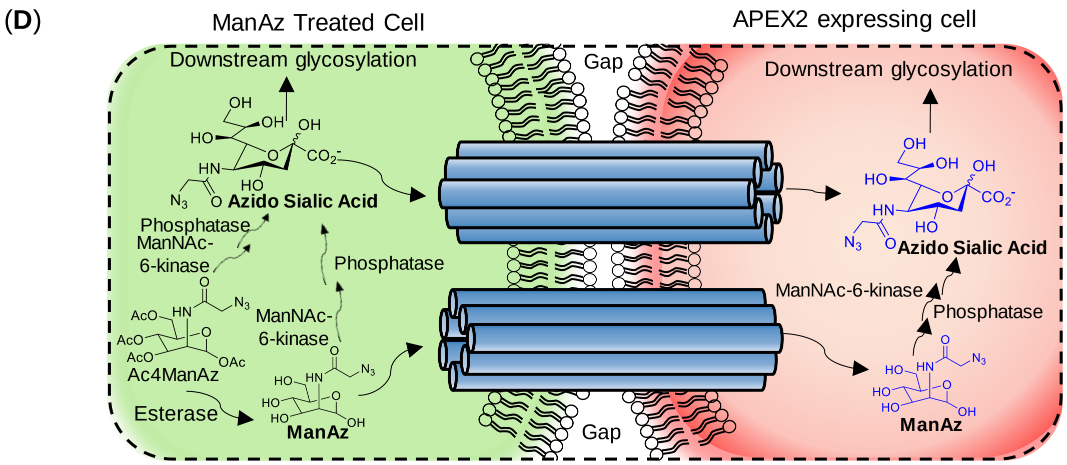

Figure R10 (revised Figure 4D). Revised schematic figure including the chemical structure of Azido Sialic acid

(Reviewer #1-9) There are far too many acronyms in this study. It was quite difficult to read as I was constantly checking what each meant. I would suggest that at the very least, DTB just be written out as desthiobiotin.

(**Response**) We thank the reviewer for this correction! We have updated the manuscript and acronym DTB has been replaced by desthiobiotin in most of our manuscript I. Thank you for this correction!

(Reviewer #1-10) Figure s4: I assume that TM-HRP has a myc tag? That isn’t clear from the caption or figure.

(**Response**). Yes, it has a myc tag and we have added this information in the caption of the figures of manuscript.

Page 2 line 39 “conjugated proteins a streptavidin-HRP” should this be via

(**Response**) Thank you for this correction! We have corrected this error as “We found that BTTAT-modified BSA generated numerous desthiobiotin-conjugated proteins via streptavidin-HRP western blotting.”

Figure 3C: Ascorbate should read NaAsc or Sodium Ascorbate or Na Ascorbate (change throughout).

(**Response**) We have used “sodium ascorbate” throughout the manuscript in the revised manuscript and we used “ascorbate” in the revised Figures.

Figure 3D. DPB lane is shown twice. H2O2 should be superscripted (fix super/subscripts throughout)

(**Response**) Thank for this correction! We have corrected these errors in our manuscript.

Figure S6: are the images mixed up here? Images show sample 1 without and GFP but the legend suggests Sample 1 should have all components. Authors need to check this. Further, there are many small errors in formatting and spacing in the figure.

(**Response**) Thank you for pointing this out, we have corrected the error in previous Figure S6. Further, anti-HA has been corrected to Frankenbody and DTB-alkyne to desthiobiotin-alkyne.

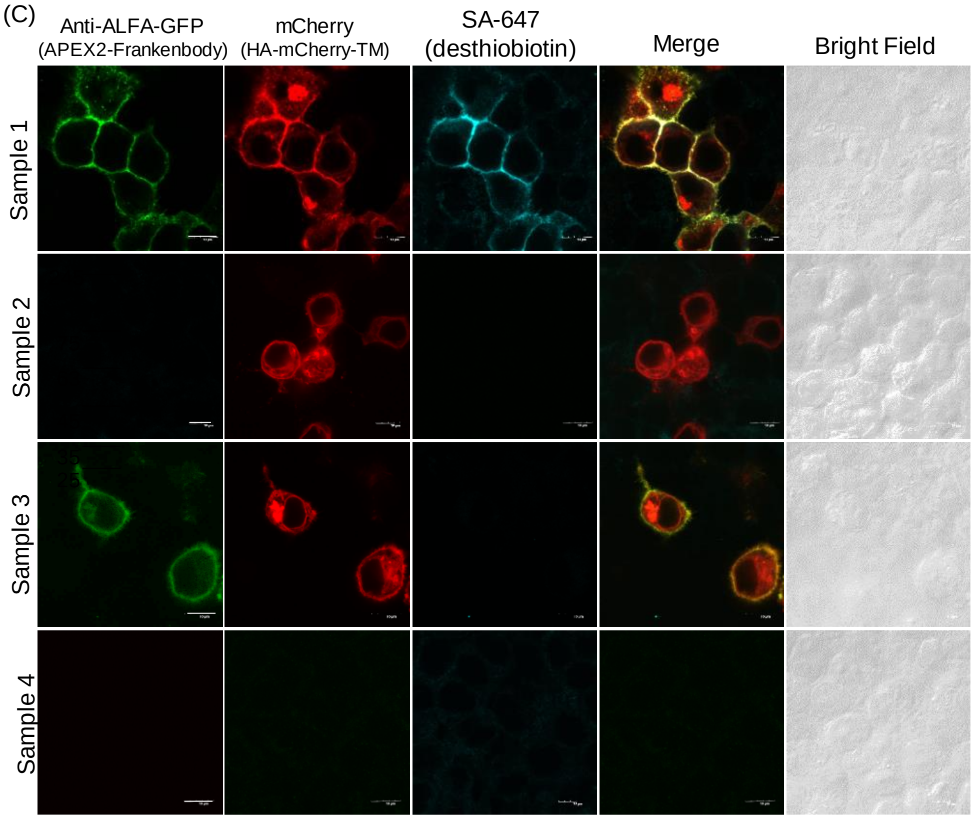

Figure R11. Corrected **Figure S6C** (Currently, it is **Figure S5C** in the revised manuscript)

Figure S6: the biotinylation is very weak in the positive lane. Can this be explained?

(**Response**) We would like to thank the reviewer for this observation, since there was only one extra band visible corresponding to receptor; however, when compared with traditional APEX2 labeling, we observed significantly enhanced and vigorous labeling, suggesting that even weak labeling with GEN-Click can result in a much shorter labeling radius (see **Figure R2** for more details).

In the procedures: “Milipore” should be Millipore

(**Response**) We have corrected it as suggested.

Additional Questions:

Quality of experimental data, technical rigor: High

Significance to chemistry researchers in this and related fields: High

Broad interest to other researchers: Top 5%

Novelty: Top 5%

Is this research study suitable for media coverage or a First Reactions (a News & Views piece in the journal)?: No

Reviewer: 2

Recommendation: Reconsider after major revisions noted.

Comments:

This manuscript by Mishra et al. describes a novel strategy to perform the CuAAC reaction in living cells with a genetically encoded tag. Such a spatially restricted click reaction benefits the profiling of newly synthesized proteome and post-translational modifications (PTMs) in a subcellular specific manner. Overall, the idea is inspiring and the authors have provided experimental data demonstrating the feasibility of this novel approach. The following issues need to be addressed before the manuscript could be considered for publication at ACS Central Science.

1. (**Reviewer #2-1**) The authors first introduced a HaloTag-based strategy, but quickly dismissed the idea because the catalytic efficiency appeared low, which the authors attributed to the "**the proteinaceous environment** of HaloTag" that "inhibits BTTA catalytic activity or enforces a mononuclear state of Cu-BTTA". However, such statement is not supported by experimental evidence. Further, the remaining parts of the manuscript focus exclusively on the peroxidase-based strategy, which is conceptually quite different from the HaloTag approach. Such a transition in the main text could be confusing to the readers. It is this referee's opinion that the HaloTag part should be removed from the main text.

(**Response**) Thank you for your comment. We appreciate your point, which aligns with the feedback provided by Reviewer 1 (#1-7). We believe that our X-ray protein crystal analysis of HaloTag-BTTA (PDB ID: 8J1O) serves as experimental evidence, demonstrating the absence of a bound copper ion near BTTA (**Figure R12**). Additionally, we observed multiple intermolecular interactions between the triazole ring of BTTA and surface residues of the HaloTag protein, such as pi-pi interactions and hydrogen bonds. These observations from our crystal structure indicate that <u>some specific residues at the HaloTag surface</u> <u>strongly inhibits the copper complexation of BTTA</u>. We believe that this knowledge could be valuable for designing a new HaloTag protein scaffold in the development of a new GEN-Click approach. While we believe that our HaloTag-BTTA X-ray crystal structure remains valuable and inspiring to scientists in the field of biocatalyst or metalloenzyme development, we are open to following the decision of the editor or reviewer if the removal of the HaloTag section is suggested.

(A) Previous (B) Updated

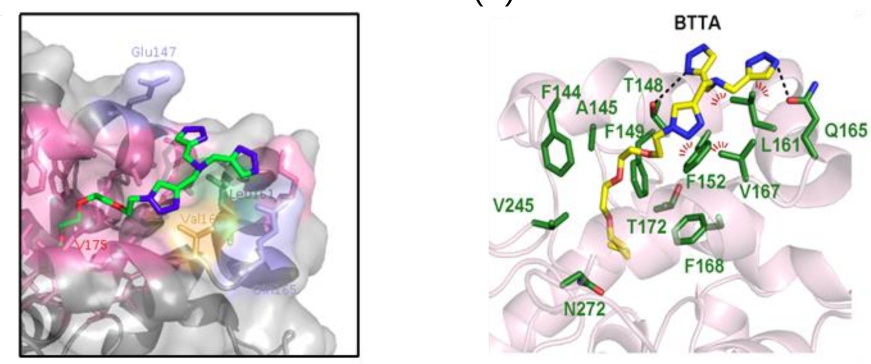

X-Ray crystal of BTTA conjugated HaloTag X-Ray crystal of BTTA conjugated HaloTag

Figure R12. Crystal structure of HaloTag-BTTA (PDB ID: 8J1O): (A) previous version (B) revised version. The newly added picture shows pi-pi and hydrogen bond interactions of BTTA moiety with surface residues.

2. (**Reviewer #2-2**) The authors report the observation of catalytic activity toward click reaction in "non-modified BSA" lacking the BTTA ligand (Figure 2B, brown trace). The authors should elaborate on the reason behind this background catalysis. Do these data suggest that free copper ions are capable of catalyzing the click reaction? There is a clear dose-dependence in the catalytic efficiency. What is the catalytic activity in the absence of BSA? More experimental data with additional controls are needed to clarify this issue.

(**Response**) We would like to thank the reviewer for the valuable comment. We agree with you. As demonstrated in our study, BSA-Cu showed higher copper-click reactivity than "No Ligand/No Cu" condition in a dose-dependent manner, indicating that BSA-Cu can effectively catalyze the copper-click reaction. In addition, we tested free Cu (no BSA) and it showed very low copper-click activity, which further strengthen the evidence of BSA-Cu can facilitate the click reaction as shown in **Figure R13**.

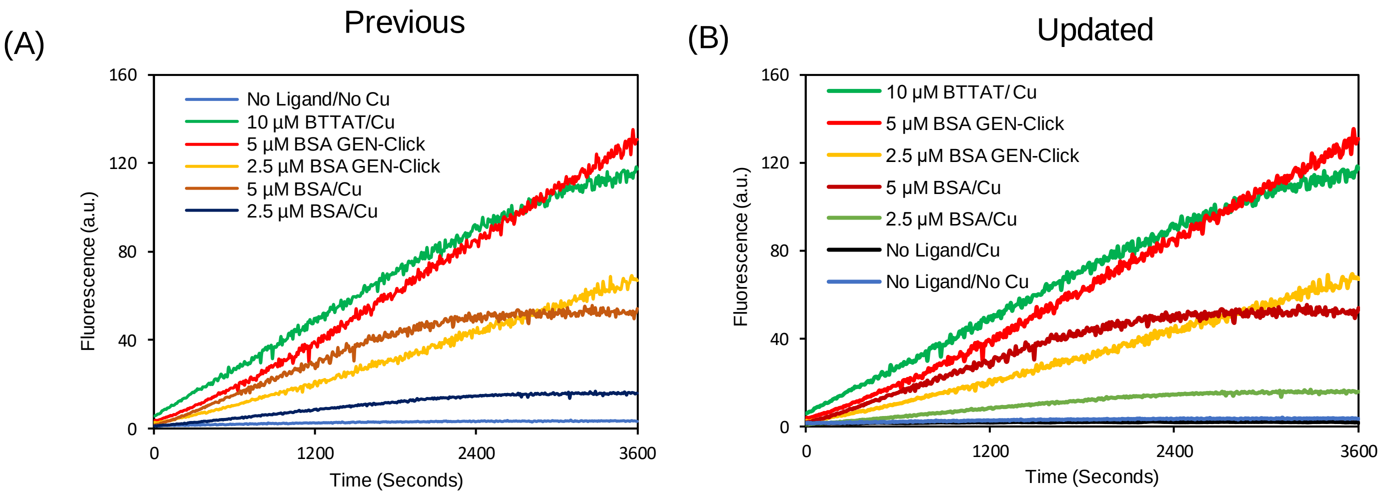

Figure R13 (related to Figure 2B) (A) previous Figure 2B and (B) updated Figure 2B with addition of no ligand/Cu condition.

We found multiple references indicating that BSA can have a copper binding site and have cited one of these references with additional comment on this event in our main text. Again, we appreciate the insightful comment of the reviewer.

"*Although basal activity of BSA with copper might be due to the metal binding sites present in the BSA*[17]"

[17] Peters, T.; Blumenstock, F. A. Copper-binding Properties of Bovine Serum Albumin and Its Amino-terminal Peptide Fragment. *Journal of Biological Chemistry* **1967**, *242* (7), 1574-1578.

3. (**Reviewer #2-3**) Page 2, line 49: with the similarity in chemical structures of BTTAT and the commercially available ligand BTTAA, why is BTTAT compared with THPTA instead of BTTAA?

(**Response**) Thank you for the valuable comment, we have updated the data comparing our ligands with BTTAA as shown in **Figure R14** (**Figure S2B**). The results indicate that BTTAA exhibited the highest activity owing to its superior water solubility. However, both BTTAT and BTTAT-PEG-HTL ligands displayed comparable activity in this assay.

(**B**)

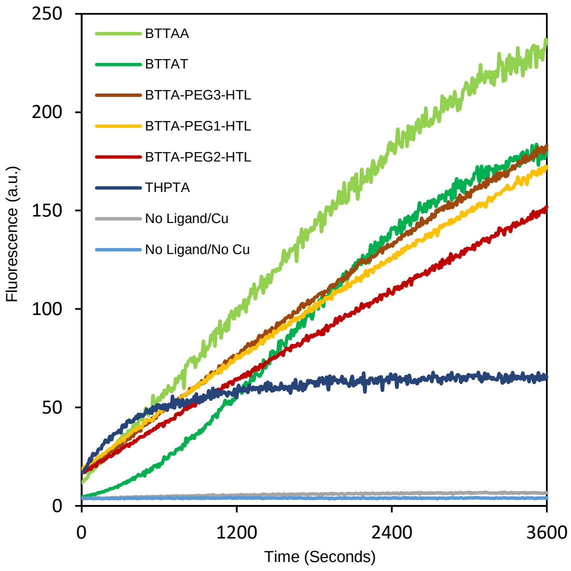

Figure R14 (related to Figure S2B). *In vitro* click reaction monitoring using 50 µM BTTA-PEGn-HTL, BTTAA and THPTA; and using 10 µM $CuSO_4$, 50 µM azidocoumarin, 100 µM propargyl alcohol, and 2.5 mM sodium ascorbate.

4. (**Reviewer #2-4**) To better demonstrate this GEN-Click system as an efficient platform for examining the metabolite-transferring events, the authors are suggested to provide at least one more metabolite-transferring case detected by the GEN-Click system.

(**Response**) Thank you for the insightful suggestion regarding the other metabolite transfer experiment. Taking your recommendation into consideration, we conducted the experiment involving transfer of Alkyne-Choline, and the result was also promising (**Figure R15**), as GEN-Click effectively captured the transfer of Alkyne-Choline via cell-cell contact. Since this comment overlaps with the comment of Reviewer 1 (#1-3), please refer to response #1-3 for more detailed information about this experiment.

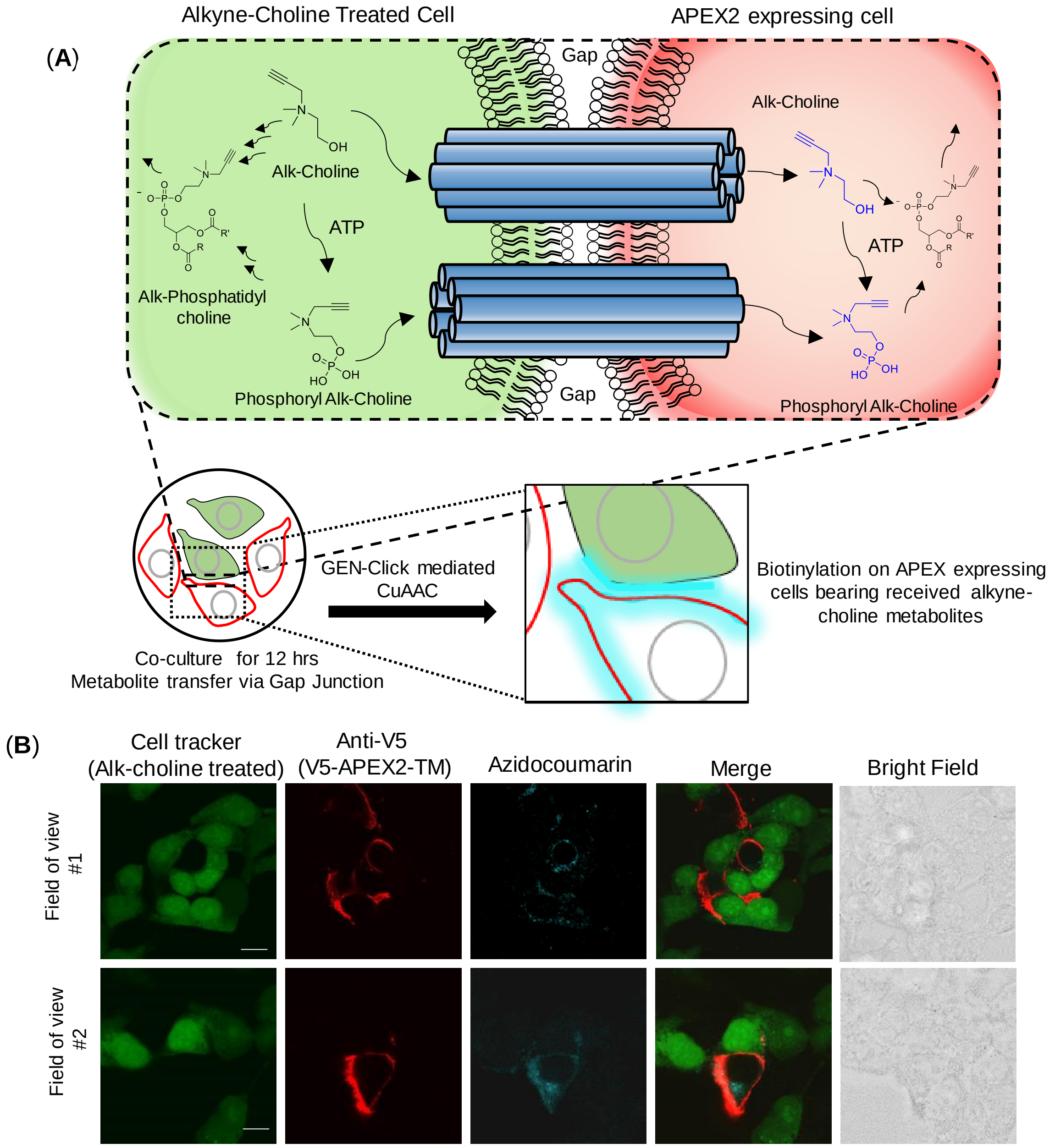

Figure R15 (related to Figure S7) (**A**) Scheme of fluorescence imaging of alkyne-choline transferring event at the cell-cell contact site using GEN-Click (**B**) Confocal images of Alkyne-Choline metabolites being transferred to adjacent contact cells in a co-cultured condition. Expression levels of APEX2-TM confirmed by anti-V5/mouse-AF568 antibody. BTTAT-Cu labeled Alkyne-Choline visualized with copper-clicked azidocoumarin (Ex=404/Em 477). Scale Bar 10 µm.

Additional Questions:

Quality of experimental data, technical rigor: High

Significance to chemistry researchers in this and related fields: High

Broad interest to other researchers: High

Novelty: Top 5%

Is this research study suitable for media coverage or a First Reactions (a News & Views piece in the journal)?: No

oc-2023-00511c.R2

Name: Peer Review Information for "GEN-Click: Genetically Encodable Click Reactions for Spatially Restricted Metabolite Labeling"

Second Round of Reviewer Comments

Reviewer: 1
Comments to the Author
The authors have done a phenomenal job addressing the reviews, adding significant data and clearing up a lot of the minor issues brought up by the reviewers.

I would still like to see the halo-tag experiments removed from the manuscript to improve the reading experience and tighten up the story. However, at this point, with all scientific issue being addressed, it is an editorial decision.

Reviewer: 2
Comments to the Author
This authors have satisfactorily addressed my concerns and comments. I have no further comments and support publication of this work.

Author's Response to Peer Review Comments:

SEOUL NATIONAL UNIVERSITY
Department of Chemistry
Seoul, 08826, Korea

HYUN-WOO RHEE, Ph.D.
Associate Professor
Department of Chemistry,
Seoul National University,
TEL. +82-2-880-4390
rheehw@snu.ac.kr

July 10, 2023

Prof. Editor
Editor
ACS Central Science

Dear Prof. Editor,

Thank you for accepting our manuscript titled “GEN-Click: Genetically Encodable Click Reactions for Spatially Restricted Metabolite Labeling” for publication in *ACS Central Science*, we were delighted to receive positive comments from both reviewers who recommended, "Publish in ACS Central Science without change."

We have prepared the final version of our manuscript as requested by editor:

1. We have removed the highlighting from the text of both the Manuscript and Supporting Information files, and upload “clean” copies for publication.
2. We have numbered references individually, with only one citation per reference.
3. The supporting information has been formatted with a cover sheet listing authors, author affiliations, corresponding author email, manuscript title, and a table of content is provided with the number of pages corresponding to figures, tables. Scheme, and synthesis protocol.

We hope that you will find the revised manuscript much improved and worthy of publication in *ACS Central Science.*

Thank you for your consideration. I look forward to hearing from you.

Sincerely,

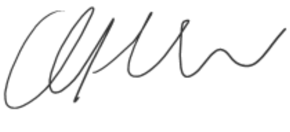

Hyun-Woo Rhee
